# A Quest for the Mechanism of Ultrahigh Resolution SEM Imaging

**DOI:** 10.1002/advs.202516341

**Published:** 2026-01-26

**Authors:** Hao Tian Chen, Y.B. Zou, B. Da, Z.J. Ding

**Affiliations:** ^1^ Hefei National Research Center for Physical Sciences at the Microscale and Department of Physics University of Science and Technology of China Hefei Anhui P. R. China; ^2^ School of Physics & Electronic Engineering Xinjiang Normal University Urumqi Xinjiang P. R. China; ^3^ Center For Basic Research on Materials National Institute for Materials Science Tsukuba Ibaraki Japan

**Keywords:** in‐lens detector, Monte Carlo simulation, secondary electron emission, SEM image, ultrahigh resolution

## Abstract

The long‐standing view in scanning electron microscopy (SEM) holds that SE1 electrons determine image resolution, whereas SE2 electrons contribute mainly to the background. However, the quantitative relationship between SE1/SE2 emission and spatial resolution has never been rigorously established due to the lack of explicit definitions for SE1 and SE2 for a Monte Carlo simulation. In this study, we develop a comprehensive simulation framework with unambiguous SE1/SE2 definitions to investigate secondary electron emission from typical solid materials by a 0.1–30 keV primary beam. The results reveal that SE1 and SE2 exhibit no intrinsic difference in emission characteristics except for the spatial spreading, and the local 3D morphology of the surface shall modulate SE2 spatial emission. Simulations for Au nanoparticles on a carbon substrate illustrate that the morphology‐modulated SE2 electrons near particle edges contribute significantly to sub‐nanometer resolution (∼0.8 nm); the exclusion of SE3 by the in‐lens detector also plays a role in resolution enhancement. This work overturns the conventional consideration that SE2 degrades resolution, and provides a unified physical explanation for the mechanism of ultrahigh‐resolution SEM imaging.

## Graphical abstract

Using Monte Carlo simulations with explicit definitions of SE1 and SE2 align with a cascade secondary electron production model, this work shows that the 3D nanostructure modulated SE2 emission enhances other than to reduce topographical contrast via a “miniature Faraday cup” effect. This explains the mechanism of sub‐nanometer resolution SEM imaging.

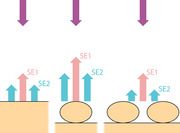

## 1 Introduction

When an electron beam strikes a solid sample in a scanning electron microscope (SEM), electron signals are emitted from the material. From a practical point of view [1], these emitted electrons with their energies higher or lower than 50 eV are defined as backscattered electrons or secondary electrons, respectively. Backscattered electrons are basically those of incident electrons which lost some of their incident energies during their penetration course from the surface into the interior of the material and then escaped from the surface; they carry the information related mostly to the atomic composition gained from elastic scattering events. Secondary electrons are mainly the excited electrons from the valence band within short depths of ∼1 nm beneath the sample surface [2], making them ideal signals for imaging of surface structure for the topographical dependence of escaping probability. These secondary electrons emitted from the sample are generally categorized into two types, i.e., SE1 and SE2 [3, 4, 5, 6, 7]. SE1 are defined to be those produced by the incident electrons at the immediate vicinity of the beam impact position, while SE2 are those produced by backscattered electrons during their course of travel to the surface. Because of the shallow emission depth of secondary electrons, the SE2 are produced near the surface when backscattered electrons reaching to the surface at a lateral distance far away from the incident location. Therefore, SE2 exhibits a significantly broader emission range compared to SE1, and SE1 is thus by far considered to be the dominant component of high‐resolution SEM imaging signals [5].

The spatial resolution of SEM continues to be a subject of ongoing research. It is of considerable interest for the instrument development. The introduction of the field emission gun has facilitated the generation of a highly focused electron beam with high intensity [8], which contributed largely to the realization of sub‐nanometer resolution SEM imaging in 1987 [9, 10]. The modern high‐resolution field emission SEMs have applied a similar system composition, i.e. a field emission cathode for reduction of beam size [11], an immersion objective lens for reduction of the aberration coefficient whose strong magnetic field can reach the specimen surface [12, 13, 14, 15], and an in‐lens or “through‐the‐lens” secondary electron detector [7, 16, 17, 18]. More recently, developments in high‐resolution SEM have also benefited from machine‐learning‐assisted reconstruction techniques, which have been used to suppress noise and enhance fine image features [19, 20, 21].

But the physical mechanism for sub‐nanometer resolution has remained unclear yet. It has been widely acquiesced that SE2 provides a slowly varying background to the SE1 signal, reducing the high‐resolution image contrast but giving a transparent appearance to the image [7, 22, 23, 24]. Therefore, it was believed that the sub‐nanometer resolution should be originated from the highly localized interaction volume of SE1. However, such a consideration involves a certain conflict with other facts in practical high‐resolution SEM observations. This is because the wide emission of SE2 from the nearby surface structures would certainly blur the emission property of the local structure that the beam is landing, particularly by considering SE2 occupy much more signal abundance than SE1 [25], if SE2 were not excluded. When signal electrons for an image pixel corresponding to the particle gap are still flooding out from the surrounding particles, then how can the pixel intensity of the gap be greatly smaller than the pixel intensity of the particle? To avoid the conflict, there was a guess that the in‐lens detectors possess different detection efficiencies between SE1 and SE2 signals because their emission properties may differ [16] although it is also generally considered that SE2 signals are indistinguishable from the SE1 based on their energy and angular distributions [7, 26].

To understand the underlying mechanism of the ultrahigh‐resolution SEM imaging, the quantitative characteristics about SE1 and SE2 must be known (not only for a planar surface but also for the nanostructures used in imaging). Although secondary electrons have been studied for decades, such full quantitative information about secondary electron emission has not been derived yet so that researchers frequently refer to the conceptual understanding about SE1 and SE2 for planar surfaces. It is known that [26, 27] the resolution limit of SEM not only depends on the inevitable aberrations and diffraction‐limited beam size [28] but is also critically related to the interaction volume [29] and, hence, many experimental factors from the electron beam, sample to the detector for the resolution measurement. In this respect, theoretical simulation can enable us to isolate the dominant influencing factors and reveal the intrinsic emission behaviors of SE1 and SE2 without complications in experiments. These intrinsic properties form a base for understanding how sub‐nanometer resolution is achieved on surfaces with 3D geometries.

Monte Carlo simulation methods have long been recognized as indispensable tools for exploring the complex physics of electron‐solid interactions, and they now underpin a wide range of electron spectroscopy and electron microscopy techniques. Early community efforts to establish Monte Carlo applications to electron probe microanalysis and SEM imaging demonstrated the value of stochastic electron trajectory modeling for clarifying electron escape mechanisms and signal formation [30, 31, 32, 33, 34, 35]. Subsequent theoretical works on electron backscattering [36, 37] and secondary electron generation [38, 39, 40, 41] have expanded Monte Carlo models of electron‐solid interactions by embedding more realistic scattering physics. The ability to model SEM signal formation in full three dimensions was later explored more rigorously for non‐planar geometries, photomasks, and critical dimension metrology [42, 43, 44]. More recent studies have advanced these approaches by incorporating electrostatic effects, showing that charging of insulating features can significantly modulate secondary electron emission and influence SEM image contrast [45]. In particular, Monte Carlo‐based libraries enable quantification of 3D critical dimensions and shape extraction at the sub‐10 nm scale [46, 47]. These techniques demonstrate that traditional edge detection methods can introduce systematic bias, and that full physical modeling is required to achieve nanometer‐level repeatability and accuracy in SEM metrology [48, 49]. Further improvements in simulation fidelity have been achieved by evaluating the impact of the first‐principles physics models. For instance, including quantum mechanical transmission and acoustic phonon scattering is crucial for an accurate study of low‐energy secondary electron emission and topography‐dependent contrast [49]. Similarly, low‐energy SEM techniques highlight the importance of quantum effects, crystallography, and charging, which classical Monte Carlo approaches alone cannot fully capture [50]. These advances collectively underline the need for flexible, modular Monte Carlo frameworks capable of modeling arbitrary 3D geometries, beam conditions, and material properties while including charging and detailed secondary and backscattered electron physics [45].

These foundational works established the numerical basis for predicting backscattering, secondary electron generation, and charge transport in solids, and they provided the conceptual framework for many of the modern simulation platforms used today. Building on this broad foundation, we have systematically developed a high‐precision Monte Carlo simulation framework based on the classical trajectory Monte Carlo (CTMC) method. A key distinguishing feature of our model is the use of Mott's relativistic formulation for electron‐nucleus elastic scattering [51], and an inelastic scattering cross‐section derived from the dielectric functional formalism [41, 52]. These choices enable accurate modeling of electron trajectories over the full energy range relevant to SEM, including the low‐energy regime critical for secondary electron emission. Our CTMC approach has been successfully applied to a variety of microscopy and spectroscopy problems, including SEM imaging of samples with complex 3D geometries [53, 54, 55, 56], Auger electron spectroscopy, and reflection electron energy loss spectroscopy [57, 58, 59]. Importantly, the reliability of the model has been validated extensively through comparison with experiments. Quantitative agreement has been demonstrated for absolute secondary electron yields and backscattering coefficients across a broad range of elemental materials [40, 60, 61, 62, 63, 64, 65], as well as for the energy distributions of backscattered electrons in Auger electron spectroscopy [66, 67].

Monte Carlo models have been employed to calculate SE1 and SE2 in some studies [68, 69] by utilizing the Rutherford elastic scattering cross section and the Bethe stopping equation, which are basically invalid at the low energies of secondary electrons. In addition, the unambiguous definition of SE1 and SE2 electron trajectories was not explicitly given in the available literatures, possibly due to their simple modeling of secondary electrons. That is, a certain number of secondary electrons were produced according to the stopping power, which were then treated by a diffusion process. Thus, the individual scattering character together with the cascade production process of secondary electrons [33, 70] has not been taken into account in these studies. The cascade process is essential to the simulation of realistic physics processes of production, transport, and emission for true secondary electrons, and it guarantees the reasonable calculation of secondary electron yield. However, including the cascade process causes difficulty to distinct the SE1 and SE2 in a Monte Carlo simulation. Lack of precise definition of SE1 and SE2 in tracking of electron trajectories will lead to doubts on the obtained quantitative data. Therefore, a precise definition for SE1 and SE2 is essential for providing a solid foundation of quantitative image analysis.

We will present three definitions of SE1 and SE2 in this work. Because the examination of the radial, lateral, depth, energy, and angular distributions of secondary electron emission holds paramount significance for the investigation of SEM resolution, we have employed the latest Monte Carlo model based on an individual inelastic scattering approach (CTMC‐SEM) [61] and incorporated the new, explicit, and computationally traceable definitions of SE1 and SE2. This formulation enables quantitative cross‐comparison among the definitions—something not previously achievable in existing simulations. Monte Carlo simulations have been performed for several typical elemental materials with primary energies from 0.1 to 30 keV, while only the elemental data related to the image resolution measurement will be presented for the sake of brevity. It is found that there is no essential difference between the two types of electrons, SE1 and SE2, on emission, except for their quantitative radial and lateral distributions. To address the inconsistency between the calculation results and the conventional interpretation of ultrahigh resolution, the Monte Carlo simulations of line‐scan profiles have been performed for nanosized gold particles on a gold substrate. The results indicate that not only the SE1 are signals in a sub‐nanometer resolution image, but SE2 also contribute the majority. The particle morphology, which demonstrates for the first time, modulates the SE2 emission and governs ultrahigh‐resolution image contrast.

## 2 Secondary Electron Classification

In our Monte Carlo model, an emitted secondary electron is classified as SE1 or SE2 based on how the electron is cascading, i.e., the complete history of its generation, including the trajectory of the parent electron and all preceding scattering events. This means that the SE1/SE2 identification is performed at the level of individual electron trajectories, rather than through indirect criteria such as energy thresholds. In the following context, the term *n*th‐generation is used to denote the family hierarchy of secondary electrons, i.e., a primary electron (i.e., zeroth‐generation) produces the first‐generation electrons (secondary electrons), and a first‐generation electron produces the second‐generation electrons (tertiary electrons) during electron inelastic scattering events. The production of electrons is actually the electronic excitation of electrons from the core shell or valence band.

### 2.1 Definition‐1 and ‐2

**FIGURE 1:**
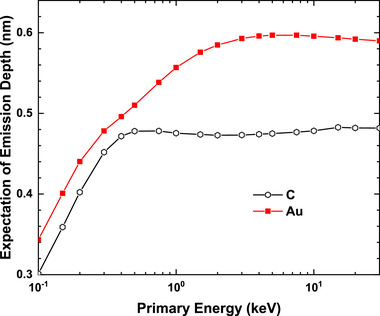
The expectation of emission depth, $\bar{d}$, for C (graphite, black) and Au (red).

In Auger electron spectroscopy, signals come from the excited Auger electrons in the surface region directly by the incident electrons and those excited by backscattered electrons returning to the surface [71], where the surface region can be defined as the depth of the electron inelastic mean free path (IMFP). A similar concept may be extended to the definition of SE1 and SE2. However, unlike Auger electrons, secondary electrons undergo cascade processes that allow even those generated deeper in the material to escape. Therefore, defining SE1 and SE2 in the present case requires introducing a reasonable thickness of the concerned surface layer. Let $d_{i}$ be the depth beneath the surface where the *i*th emitted true secondary electron signal is generated. The averaged depth, $\bar{d}=\frac{1}{N}\sum_{i=1}^{N}d_{i},$ where *N* is the total number of secondary electrons, is ∼1 nm beneath the sample surface, depending on material and primary energy [2], as shown by Figure 1. In the definitions‐1 and ‐2, the thickness of the surface layer responsible for SE1 generation is taken to be $3\bar{d}$, to exclude secondary electrons originating from the deeper regions of the sample. For the materials and beam energies considered here, $\bar{d}$ ranges from 0.3 to 0.6 nm, giving a corresponding surface‐layer in thick of ∼1–2 nm.

If we trace the trajectory of a true secondary electron signal back to that of the zeroth‐generation electron, we can know where the first‐generation electron is produced, and the moving direction of the zeroth‐generation electron at that location. Let $d^{\left(1\right)}$ be the depth of the first‐generation where it is produced, and $\theta^{\left(1\right)}$ be the angle between the primary beam incident direction and the zeroth‐generation electron moving direction at the location where the first‐generation electron is produced. We also denote $d^{\left(f\right)}$ to be the emission depth of the true secondary electron. Then the definitions of SE1 and SE2 are given as:

1. If a primary electron is emitted with energy lower than 50 eV, then it is SE1;
2. The secondary electron is SE1 if $d^{\left(1\right)}<3\bar{d}$, $d^{\left(f\right)}<3\bar{d}$ and $\theta^{\left(1\right)}<\theta_{c}$;
3. The secondary electron is SE2 if $d^{\left(f\right)}<3\bar{d}$ except SE1;
4. Otherwise ($d^{\left(f\right)}>3\bar{d}$), it is noted as SEX.

**FIGURE 2:**
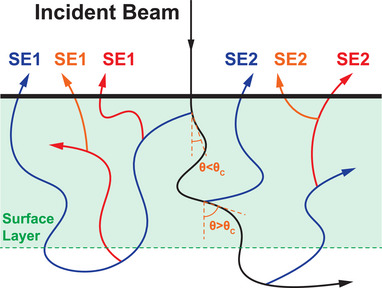
The schematic diagram for the definition‐1 and ‐2 of SE1 and SE2. The black line represents the primary electron, and the blue, red, and orange lines represent the first, second, and third generations of secondary electrons.

In the definition, $\theta_{c}$ represents the critical scattering angle, and Figure 2 shows the schematic diagram. For a primary electron to be considered backscattered, its scattering angle must exceed 90°. Accordingly, we choose $\theta_{c}$ to be 45° and 90° in definition‐1 and definition‐2, respectively, to represent a stricter SE1 criterion and a more intuitive SE1 and SE2 criterion.

### 2.2 Definition‐3

Definition‐1 and ‐2 require defining the thickness of the surface layer. However, for samples with structured or non‐planar surfaces, the surface layer cannot be clearly defined. Therefore, we introduce a third definition that does not rely on any geometric parameters. For planar surfaces, this definition yields results consistent with definition‐1 and ‐2, while remaining applicable to complex surface geometries.

Backscattered electrons are defined either as the emitted electrons signals originated from incident electrons which suffer at least one high‐angle (>90°) scattering event [72], or as those electrons having energies greater than 50 eV. However, neither definition offers a practical identification of SE2. The qualitative picture of backscattered electrons may be given visually, considering that a high‐angle scattering event means the electron moving direction is turning backward toward the surface.

Let us image the whole course of the path of an emitted true secondary electron from its birth location back to the incident position of the primary electron as the ancestor of this true secondary electron. The shape of this path is not important, but two numbers are. The one is the maximum depth, *d*_max_, of this path, and the other one is the depth of the ending position, *d*_end_, of the path. Note that *d*_end_ is exactly the birth depth of the true secondary electron signal, therefore, the definition‐3 of SE1 and SE2 is given as:

1. If a primary electron is emitted with energy lower than 50 eV, then it is SE1;
2. If *d*_end_ = *d*_max_ for a true secondary electron, then it is SE1;
3. Otherwise, all the other true secondary electrons are SE2.

**FIGURE 3:**
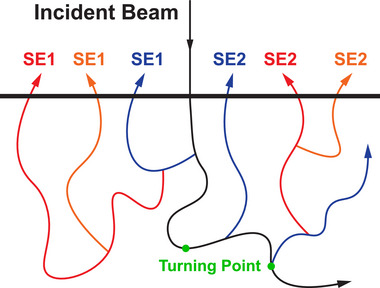
The schematic diagram for the definition‐3 of SE1 and SE2. The black line represents the primary electron, and the blue, red, and orange lines represent the first‐, second‐ and third‐generations of secondary electrons.

By this definition, we only consider the path shape when *d*_end_ = *d*_max_, the overall path shape of the primary electron trajectory is moving forward into the solid, and the excited secondary electron is thus SE1. If *d*_end_ < *d*_max_, then the path turns to the backward direction of incidence, and the secondary electron is attributed to SE2. Figure 3 illustrates the schematic diagram of definition‐3, which is more analogous to definition‐2 except that no surface layer is involved here.

## 3 Results and Discussion

We have performed Monte Carlo simulations for primary electrons normally incident onto the ideal smooth and clean surfaces of C (graphite) and Au at first. The calculated primary electron energy is ranged from 0.1 to 30 keV, and for each primary energy 1 × 10^8^ primary electron trajectories were employed. Information of the emitted electrons is then collected.

**FIGURE 4:**
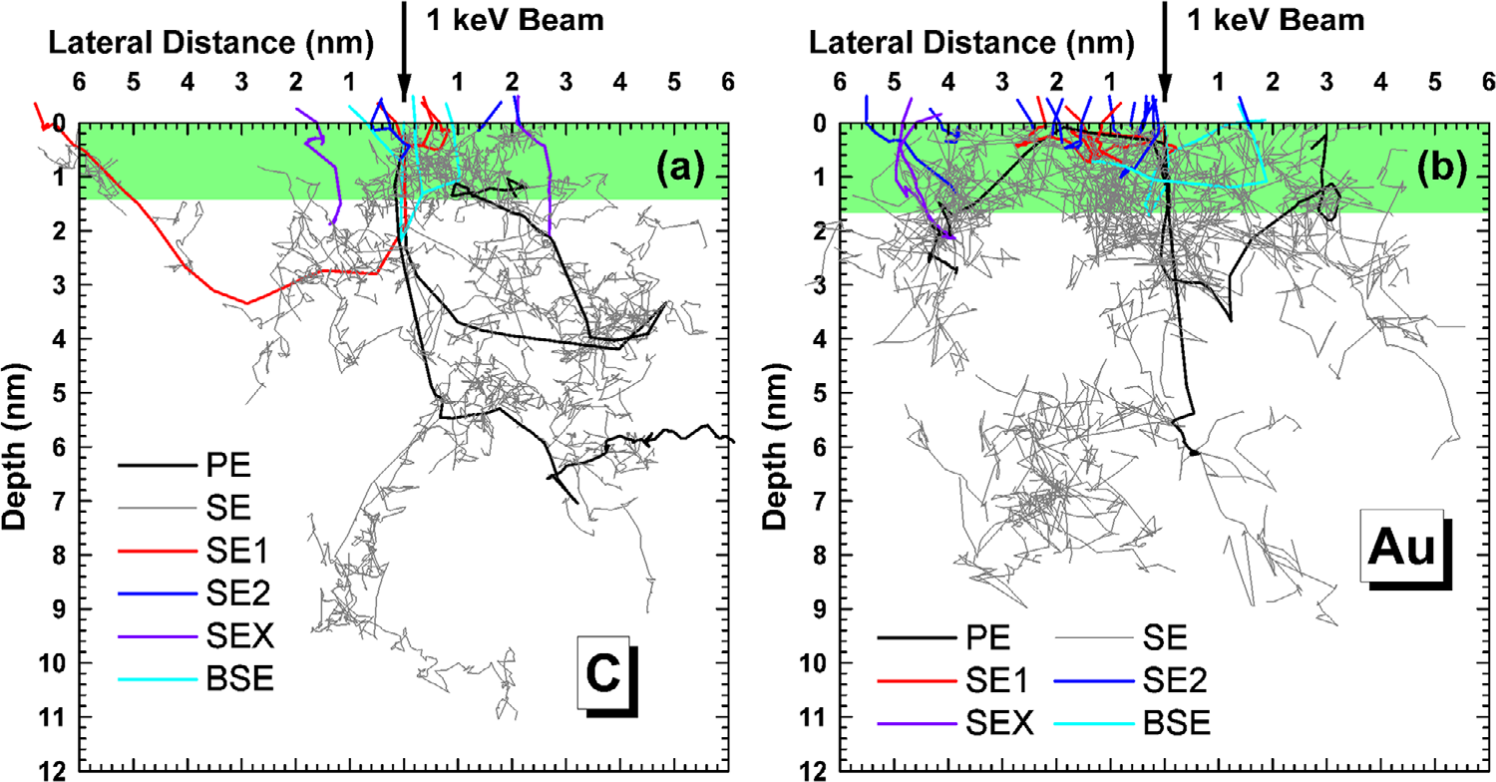
Trajectories of five primary electrons and all the generated secondary electrons by the definition‐1 in (a) graphite and (b) gold at primary energy of 1 keV, where the color of the lines represents: absorbed primary electrons (black), absorbed secondary electrons (gray), SE1 (red), SE2 (blue), SEX (violet), backscattered electrons (cyan). The green area represents the defined surface layer.

In Figure 4 the trajectories of five primary electrons and all the generated secondary electrons by definition‐1 are plotted. These trajectories can be categorized into six groups: the absorbed primary and secondary electrons; the SE1, SE2, and SEX electrons, and the backscattered electrons.

### 3.1 Radial and Lateral Distributions

From a Monte Carlo simulation, we can obtain the emission positions on the sample surface of all the emitted electrons. Let the *xy*‐plane of the Cartesian coordinate be the sample surface, and the origin be the position of the electron beam incidence. The intensity distribution of emitted secondary electrons, *I*_SE_(*x*,*y*), becomes the probability density distribution that a secondary electron is emitted at the position (*x*, *y*) on the surface plane, after normalization,

(1) $$\int I_{\mathrm{SE}}\left(x,y\right)dxdy=1$$

**FIGURE 5:**
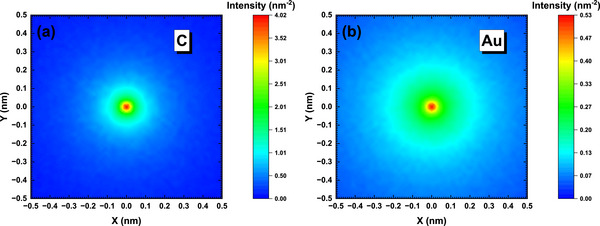
The intensity *I*_SE_(*x*,*y*) at primary energy of 5 keV for (a) graphite and (b) gold.

The distributions for C and Au at primary energy of 5 keV are shown in Figure 5. Since *I*_SE_(*x*,*y*) is symmetrical about the incidence point, the radial density distribution, *I*_SE_(*r*), is thus derived which satisfies

(2) $$2\pi \int_{0}^{+\infty }I_{\mathrm{SE}}\left(r\right)rdr=1$$

where *r* is the radial distance of the emission from the origin. Furthermore, the lateral distribution, *L*_SE_(*x*), of secondary electrons emitted at position *x* and within the incidence plane defined by |*y*| < 0.01 nm, which satisfies

(3) $$\int_{-\infty }^{\infty }L_{\mathrm{SE}}\left(x\right)dx=1$$

**FIGURE 6:**
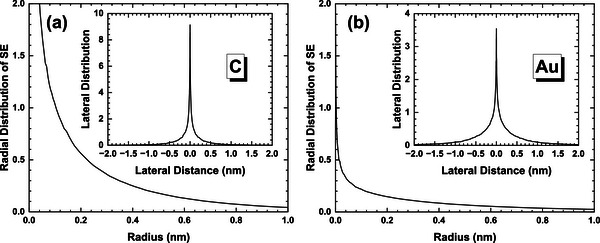
The radial distributions, *I*_SE_(*r*), at primary energy of 5 keV for (a) graphite and (b) gold. The insets show the corresponding lateral distributions, *L*_SE_(*x*).

The comparison of the calculated radial distributions *I*_SE_(*r*) and lateral distributions *L*_SE_(*x*) at primary energy of 5 keV is given in Figure 6. It shows that Au presents a slightly wider lateral distribution than that of C.

We can also obtain the normalized radial distributions of SE1, *I*_SE1_(*r*), and SE2, *I*_SE2_(*r*). Let *N*_SE_, *N*_SE1_ and *N*_SE2_ be the number of total true secondary electrons (noted as SE), SE1 and SE2, respectively, for a given number of simulated incident electrons,

(4) $$N_{\mathrm{SE}}=N_{\mathrm{SE}1}+N_{\mathrm{SE}2}+N_{\mathrm{SEX}}\approx N_{\mathrm{SE}1}+N_{\mathrm{SE}2}$$

We define the weighted radial distributions,

(5)

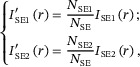

so that *I*′_SE_(*r*) ≈ *I*′_SE1_(*r*) + *I*′_SE2_(*r*). Note that the weighted radial distributions are no more the normalized probability density distributions, but these defined quantities are useful for comparing the intensity of each component.

**FIGURE 7:**
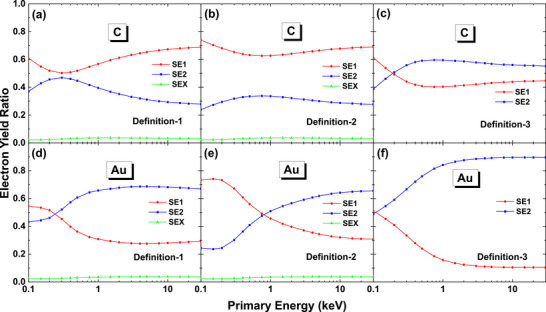
The ratio of numbers of SE1 (red), SE2 (blue), and SEX (green) to the number of SE for graphite (top row) and gold (bottom row). The left, middle, and right columns are calculated by the definition‐1, ‐2 and ‐3, respectively.

Figure 7 shows the ratio of *N*_SE1_, *N*_SE2_ and *N*_SEX_ to *N*_SE_ as functions of primary energy. It is seen that the three definitions give a similar variation trend of numbers of SE1 and SE2 with primary energy, while different definition results in different quantitative values, as it should be. In terms of the numbers, definition‐1 is between definition‐2 and ‐3. For gold and above 1 keV, *N*_SE2_ is much higher than *N*_SE1_ by either the definition of SE1 and SE2. Especially, by definition‐3 which is closer to the conventional concept of SE1 and SE2, *N*_SE2_ is about nine times of *N*_SE1_ at 10 keV; and by the definition‐2, *N*_SE2_ is about three times of *N*_SE1_. This quantitative ratio is very important in understanding the imaging signals even at sub‐nanometer resolution.

By the same step, we can also obtain each component of the weighted lateral distributions as,

(6)

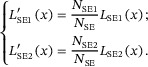




**FIGURE 8:**
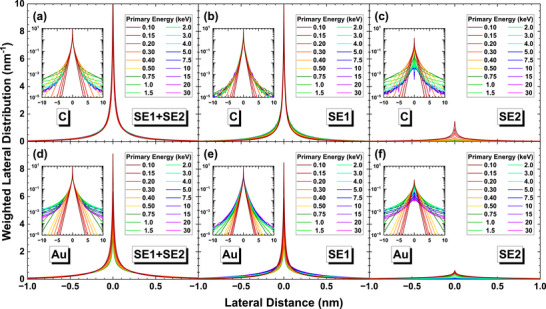
Weighted lateral distributions for graphite (top row) and gold (bottom row). The left, middle, and right columns are for SE, SE1, and SE2 components, respectively, by the definition‐1. The insets show the intensity in logarithmic scale. Colors indicate beam energy: 0.1–0.5 keV (dark red to yellow), 0.75–4 keV (dark green to cyan), and 5–30 keV (dark blue to magenta).

Figure 8 shows the weighted lateral distributions of SE, SE1, and SE2 components according to definition‐1. In these figures, the lateral distributions at different energies are nearly the same for C above 1 keV and vary obviously for Au. Notice that SE1 and SE2 have a similar distribution shape in Figure 8b,c, and it looks as if SE1 dominates the SE in amount by comparing Figure 8a,b. But this observation is contrary to the result shown in Figure 8. The insets in Figure 8 explain the reason. In fact, SE2 component has a rather wider distribution than the SE1. At a far distance from the incidence location, the intensity of SE2 emission is about one order of magnitude higher than that of SE1 by comparing Figure 8e,f for Au. Therefore, qualitatively the SE1 dominates the emission in a region of ∼1 nm around the incidence location while more SE2 are emitted far away.

**FIGURE 9:**
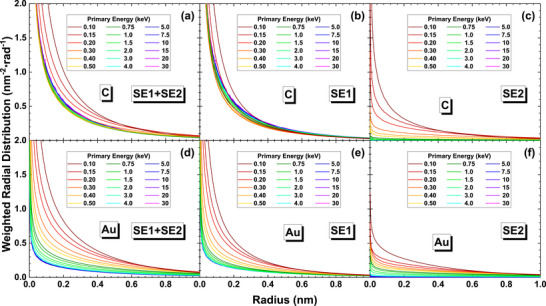
Weighted radial distributions for graphite (top row) and gold (bottom row). The left, middle, and right columns are for SE, SE1, and SE2 components, respectively, by definition‐1. Colors indicate beam energy: 0.1–0.5 keV (dark red to yellow), 0.75–4 keV (dark green to cyan), and 5–30 keV (dark blue to magenta).

Figure 9 illustrate the weighted radial distributions of SE, SE1, and SE2 components by definition‐1 (the results of definition‐2 and ‐3 are provided in Figures S1 and S2). It can be found that, as the primary energy increases, there is a corresponding decline in emission intensity near the origin. Since the emission area per unit radial interval is proportional to *r* ^2^, a large portion of electrons is emitted from the regions far away from the central area. Consequently, the variances of these radial distributions increase as the primary energy is raised. The middle and the right columns of Figure 9 display the distributions for the SE1 and SE2 components of emission. Both components inherit the properties that the weighted radial distribution of SE has, but one can clearly see the difference between them: SE1 component is much stronger than SE2 component near the origin. Figures S3 and S4 provide further the ratio between SE, SE1, and SE2. The weighted radial distributions of other definitions can be found in Appendix, which are almost identical to the distributions of definition‐1. Figure 9 also compares the results derived from three different definitions of SE1 and SE2. Clearly, there is not much difference in the curve shape and trend of energy variation between these definitions.

Another aspect of radial distribution is its quantification of the intensity near the incidence location. An object point corresponds to the size of a small local excitation volume designated as the spatial detection limit from which a sufficient signal can be obtained [16]. When *r* → 0, the radial distribution intensities increase dramatically. This trend suggests that the simple use of the full width at half maximum (FWHM) as a metric of detection limit or the theoretical resolution is not suitable. Consequently, alternative statistics are used to characterize the spatial emission properties of SE1 and SE2.

### 3.2 Radial Distribution Statistics

We then define the radius, *R*, of a circular area which includes 68% of concerned electrons, where the number 0.68 represents the fraction of probability within the range of [− σ, σ] in 1D Gaussian distribution of standard deviation of σ,

(7) $$2\pi \int_{0}^{R}I\left(r\right)rdr=0.68$$

while *R* corresponds to a circle radius of ∼1.5σ if *I*(*x*, *y*) obeys the 2D Gaussian distribution of standard deviation of σ. This *R* is usually called 68th percentile.

**FIGURE 10:**
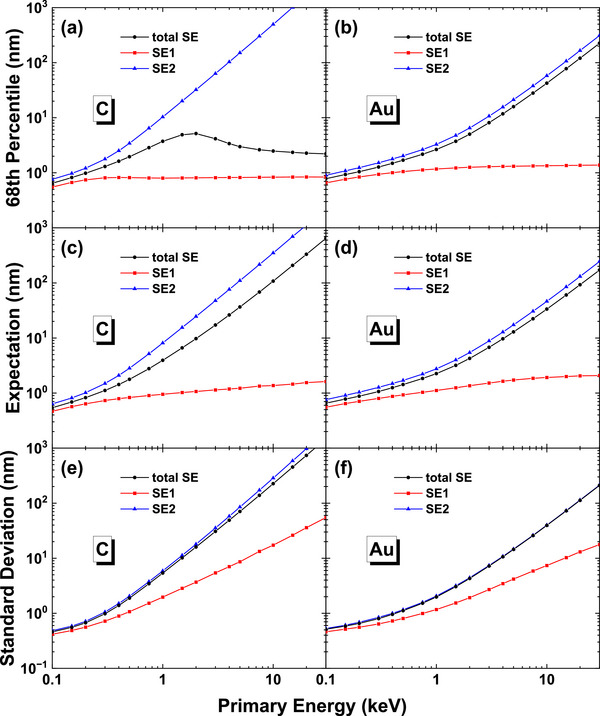
The radial statistic metrics for graphite (left column) and gold (right column) calculated using definition‐1. The top, middle, and bottom rows are the 68th percentiles *R*, the expectation values $\bar{r}$ of radial distributions, and the standard deviations σ_*r*_ of radial distributions calculated by Equations (7)–(9), respectively. Black, red, and blue curves correspond to total SE, SE1, and SE2, respectively.

The first row of Figure 10 present the calculated 68th percentiles *R* of SE, SE1, and SE2 by definition‐1. The results of other definitions are in Figures S5 and S6. The results quantify the previous comprehension about SE1 and SE2, i.e., the SE1 concentrates near the beam incident position with a constant radius of ∼1 nm at all primary energies, while the SE2 spreads more widely and has energy dependence. For C in the first column of Figure 10, the radii of SE2 increase rapidly with the primary energy. For Au in the second column of Figure 10, under all the definitions, the radii of SE2 and SE increase rapidly with the primary energy such that $R_{\mathrm{SE}}∼200$ nm at 30 keV, since SE2 dominates the secondary electron population as shown in Figure 7.

In addition to the 68th percentile, we have also calculated the expectation values, $\bar{r}$, and the standard deviationsσ_*r*_ of the radial distributions to have different measures of theoretical resolution limit,

(8) $$\bar{r}=2\pi \int_{0}^{\infty }I\left(r\right)r^{2}dr$$

(9) $$\sigma_{r}={\left[2\pi \int_{0}^{\infty }I\left(r\right)r^{3}dr-{\bar{r}}^{2}\right]}^{1/2}$$

for SE, SE1, and SE2, which are shown in the middle and the bottom rows of Figure 10, and those values of some specific energies are shown in Table 1 and Table 2 for quick reference. The standard deviations of these distributions always increase with the primary energy even for SE, and the standard deviations of SE1 are always lower than that of SE2 as expected. The expectations $\bar{r}$ and the radius *R* for SE show almost the same results. Secondary electrons are more likely to concentrate at the origin for a lower energy beam, indicating that low voltage SEM has the capability to achieve higher resolution from the point of view of beam‐sample interaction [7]. For other calculated elemental solids (Si, Fe, Cu, Ag, and Pt), the tendency of $\bar{r}$ and *R* on primary energy agrees with that of Au, demonstrating that this behavior is universal. Therefore, it is hard to explain the sub‐nanometer resolution imaging simply with the concept of SE1 and SE2. This leads to the necessity to explore the emission properties of SE1 and SE2 in every respect.

**TABLE 1:** the 68th percentiles *R*, the expectation values $\bar{r}$ of radial distributions, and the standard deviations σ_*r*_ for C (graphite) across different definitions.

Static Metric	Energy (keV)	SE	Definition‐1		Definition‐2		Definition‐3	
			SE1	SE2	SE1	SE2	SE1	SE2
*R*	0.75	2.86	0.81	6.47	0.96	6.70	0.74	5.16
	3	4.15	0.82	63.48	0.86	63.28	0.68	40.66
	10	2.48	0.83	496.8	0.84	494.4	0.67	250.6
$\bar{r}$	0.75	2.77	0.90	5.16	1.30	5.41	0.90	4.04
	3	17.21	1.14	47.55	2.52	47.53	1.04	28.98
	10	107.8	1.37	350.1	5.16	348.1	1.19	191.2
σ_*r*_	0.75	3.44	1.52	3.75	2.22	3.70	1.64	3.75
	3	30.67	5.42	35.47	11.64	35.26	5.89	35.66
	10	225.5	17.29	285.6	52.08	285.2	18.59	273.0

**TABLE 2:** the 68th percentiles *R*, the expectation values $\bar{r}$ of radial distributions, and the standard deviations σ_*r*_ for Au across different definitions.

Static Metric	Energy (keV)	SE	Definition‐1		Definition‐2		Definition‐3	
			SE1	SE2	SE1	SE2	SE1	SE2
*R*	0.75	2.17	1.13	2.64	1.57	2.68	1.09	2.40
	3	8.12	1.29	10.68	2.11	10.77	0.96	9.08
	10	42.45	1.35	57.83	1.70	57.74	0.91	47.64
$\bar{r}$	0.75	1.84	1.03	2.21	1.41	2.24	1.04	2.02
	3	6.76	1.52	8.83	3.23	8.91	1.18	7.49
	10	33.45	1.92	46.29	7.42	46.29	1.18	37.21
σ_*r*_	0.75	1.52	0.99	1.58	1.31	1.60	1.10	1.55
	3	7.20	2.70	7.37	5.29	7.36	2.43	7.30
	10	39.39	7.38	39.91	22.00	39.75	5.36	39.92

### 3.3 Emission Depth Distributions

A true secondary electron (<50 eV) will be either the energy exhausted primary electron, or an electron generated in a certain depth of a material. The former contribution is negligible except at very low primary energies. To obtain the information about the emission depth of secondary electrons, here we only consider those generated secondary electrons inside the material. Let *D*_SE_(*z*) (*z* > 0) be the depth distribution of the emitted secondary electron signals,

(10) $$\int_{0}^{+\infty }D_{\mathrm{SE}}\left(z\right)dz=1$$

The mean depth presented in Figure 1 is calculated by,

(11) $$\bar{d}=\int_{0}^{+\infty }zD_{\mathrm{SE}}\left(z\right)dz$$

Let *D*_SE1_(*z*) and *D*_SE2_(*z*) be the depth distributions of the generated SE1 and SE2 electrons, respectively. Similar to Equation (14), we have weighted depth distributions,

(12)

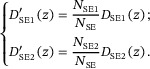




**FIGURE 11:**
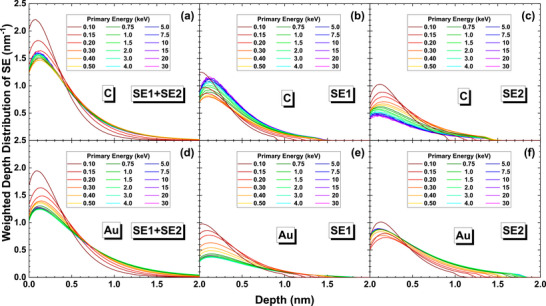
Weighted depth distributions for graphite (top row) and gold (bottom row). The left, middle, and right columns are for SE, SE1, and SE2, respectively, by definition‐1. Colors indicate beam energy: 0.1–0.5 keV (dark red to yellow), 0.75–4 keV (dark green to cyan), and 5–30 keV (dark blue to magenta).

Figure 11 presents the weighted depth distributions of SE, SE1, and SE2 components by definition‐1, other definition results are provided in Figures S7 and S8. These figures show that the maximum generation depths of SE1 have not much difference with that of SE2, and the distribution curves are similar for SE1 and SE2 while the intensity of SE1 at the surface region is somewhat stronger than that of SE2. Therefore, the emission depth should not be responsible for high‐resolution of an SEM image.

### 3.4 Angular Distributions

**FIGURE 12:**
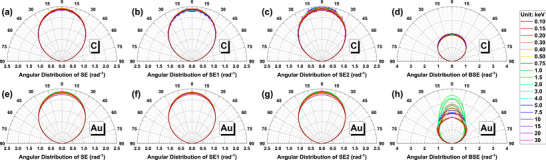
Angular distributions of graphite (top row) and gold (bottom row). The columns from left to right are for SE, SE1, SE2, and backscattered electrons, respectively. Colors indicate beam energy: 0.1–0.5 keV (dark red to yellow), 0.75–4 keV (dark green to cyan), and 5–30 keV (dark blue to magenta).

Calculated angular distributions of SE, SE1, and SE2 by definition‐1 are shown in Figure 12. It is known that angular distribution of secondary electrons obeys the cosine law. We have found that the angular distributions of SE1 and SE2, as well as that of SE are identical at all primary energies for both C and Au. The same results are obtained by the three different definitions. This result indicates that there is no noticeable difference in the emission directional characteristics between SE1 and SE2. Therefore, the emission angular property cannot provide an explanation for ultrahigh resolution SEM imaging.

For comparison, the angular distributions of backscattered electrons are given in the right column of Figure 12. For light element C, it is quite close to a cosine distribution; but for heavy element Au the distribution is deviated from the cosine law at low energies because of the strong differential elastic cross section at backward direction [73]. It seems that the in‐lens detector could be a little help for improving SEM image contrast of particles by collecting the high‐angle backscattered electrons; but this is impractical because it requires an extremely short working distance to enlarge the solid angle of detection for electron signals entering into the objective lens, and the fraction of detectable backscattered electrons to secondary electrons is quite small.

### 3.5 Energy Distributions

The calculated energy distributions of SE, SE1, and SE2 are normalized such that the area under the spectrum curve is unity in each case and are presented in Figures S9 and S10. There is no fundamental difference between SE1 and SE2 found in their energy distributions; this fact excludes the possibility to distinguish SE1 from SE2 by their energies.

### 3.6 Role of SE2

Based on the above results obtained for the planar sample surface, one can see that SE1 do not have usable distinct emission properties from those of SE2 for the improvement of spatial resolution except for the emission radius *R*. In principle the in‐lens detectors should not be able to distinguish SE1 and SE2, and the imaging signals are indeed made full of SE1 and SE2 [7]. This observation leads to the large values of *R* about a hundred nm when SE2 is included in imaging, which seems to be contrary to the experimental observation of sub‐nanometer resolution. To solve the puzzle, we need to start from the experimental method for the resolution determination of an SEM image.

**FIGURE 13:**
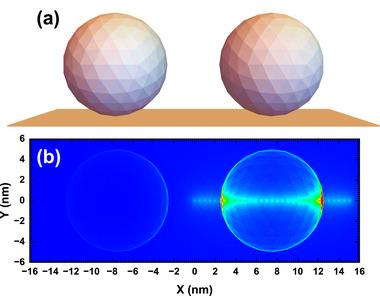
(a) Schematic diagram of two gold particles on a gold substrate. (b) Electron emission density map by collecting the emitted true secondary electrons at a primary energy of 5 keV. This map is obtained by superimposition for all simulated incident positions scanning along a line scanning over the right particle in (a).

**FIGURE 14:**
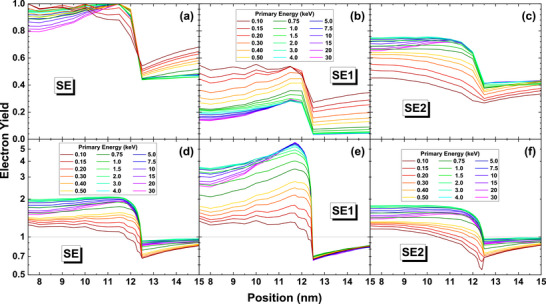
Simulated SE, SE1, and SE2 yields over the right particle in Figure 13a, with the horizontal axis defined such that the origin corresponds to the midpoint between the two spheres. Top row: at each energy, the SE yield is normalized by its maximum at the corresponding beam energy. Bottom row: at each energy, the SE, SE1, and SE2 yields are normalized by the respective ones obtained from a planar Au substrate. Colors indicate beam energy: 0.1–0.5 keV (dark red to yellow), 0.75–4 keV (dark green to cyan), and 5–30 keV (dark blue to magenta).

Practically, the gold or platinum particles on a carbon substrate have frequently been used as the standard sample for the resolution measurement because of their high and low secondary electron yields, respectively, of the respective elemental solids. We will consider how the sample structure modulates the emission property of SE1 and SE2. For this purpose, we constructed a particle/substrate sample geometry and performed the corresponding Monte Carlo simulation of secondary electron emission. Only definition‐3 is employed for particle/substrate because in this case, the surface layer is hard to define. The sample geometry consists of two gold spheres with a diameter of 10 nm with a 5 nm gap in between, and two balls are placed on the flat surface of a bulk gold substrate. The 10 nm Au particle was chosen as a typical nanoscale feature relevant to the SEM resolution test, since smaller and larger particles are usually distributed in a practical resolution sample. Here we have not employed the carbon substrate so the calculation results represent solely the structure factor without including the chemical factor. It is worth to mention that this geometry modeling causes more severe resolution estimation than the practice because the carbon substrate enables much higher contrast of the particle. The sphere surfaces are constructed by a finite element method, as shown in Figure 13a. The primary electrons incident vertically onto the sample substrate plane and scan along the line connecting the centers of the two spheres at an interval of 0.5 nm in Figure 14b. Since the sample geometry is symmetrical, we only consider the scanning over one particle and the adjacent particle is placed to present the shadowing effect. The primary energy is ranged from 0.1 to 30 keV; for each primary energy and each scanning position, 1 × 10^6^ primary electron trajectories were traced.

Figure 13b shows the electron emission density map at 5 keV by collecting the emitted true secondary electrons. It should be noted that this figure is not a usual simulated SEM image but a superimposed intensity map for scanning over the considered incident positions. It is used to enhance the understanding of the image signal contribution presented later. The bright spots at the given scanning positions of the electron beam hitting on the sphere and substrate surfaces are clearly seen, which correspond to the respective radial distributions shown in Figure 5b for the planar surface at these incident positions. However, different from the planar surface case, the particle structure has altered the emission characteristics. More SE2 signals are produced when the beam is incident closer to the protruding structure edge, as seen by comparing the spot intensity at the right edge of the sphere with that on the top surface of the sphere and that on the substrate surface. Furthermore, it is easy to find the bright intensity at the sphere edge. This is because SE2 is now more easily emitted from the sphere surface to contribute to image signals than the case of a planar surface whose boundary condition has limited secondary electron emission. Hence, SE2 is modulated by particle morphology to be a useful SEM imaging signal to enhance the particle intensity for SEM imaging of the 3D structure. This observation can help to understand the simulated line‐scan profiles over the particle presented in Figure 14. In contrast, previous understanding about the line‐scan component is that, at high magnifications of SEM imaging, the SE2 contribution only insignificantly varies from pixel to pixel, whereas the SE1 contribution depends sensitively on local features as small as the beam diameter [16].

The top row of Figure 14 shows the simulated line‐scans (the normalized electron yields) of SE, SE1, and SE2 along the scanning positions over a particle. The relative error of this simulation is 10^−3^, which is far below the visual resolution of the plots. One can see from Figure 14a that the line‐scan profile of SE is quite similar in shape to the step function,

(13) $$\Theta \left(x\right)=\left\{\begin{array}{c} 1,\;x\geq 0;\\ 0,\;x<0, \end{array}\right.$$

at an edge (*x* = 0) of a particle for an ideal incident beam with an infinitesimal probe size. It is worth noting that the line‐scan profiles of SE1 in Figure 14b clearly demonstrate the edge bloom effect for primary energy down to 200 eV. However, such an edge effect is usually found for particle sizes greater than 50 nm and is hardly observable for sizes of ∼10 nm in an experimental SEM image above several keV. For an ultrahigh resolution image of particles, the small particles do not present bloom or sharp edges either. This is strong evidence that the signals in ultrahigh‐resolution SEM imaging are not simply made of SE1 as previously supposed. Instead, the edge profile in Figure 14a is dominantly contributed by SE2 by comparing Figure 14a with Figure 14b,c. With primary energy down to 1 keV, the particle edge is still not well‐smoothened in the line‐scan of SE2. Thus, SE2 contributes significantly to the particle image contrast at high energies. The present calculation results then disprove the previous qualitative conception that at high magnifications, the SEM image resolution is determined by the spatial distribution of the SE1 signal while the SE2 signal contributes only to the background noise of the image [74].

**FIGURE 15:**
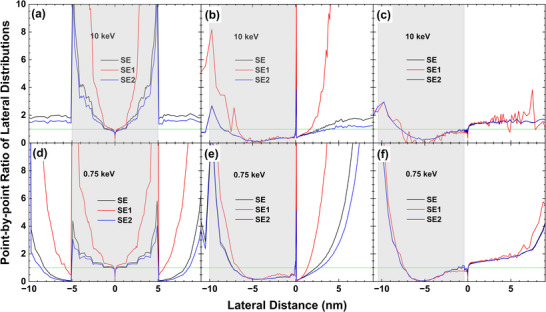
Point‐by‐point ratio of lateral distributions taken between the Au particle/Au substrate case and planar Au surface case, where the denominator of the ratio is taken from Figure 8d‐‐f. The spherical Au particle is in diameter of 10 nm. The top and the bottom rows are for 10 keV and 0.75 keV, respectively. The left, middle, and right columns are for the beam positions at the sphere top, inner side (−0.1 nm away) of sphere edge, outer side (0.4 nm away) of the sphere edge, respectively, where the origin of the horizontal axis is the beam incident position. The shaded area represents the region of the Au particle occupied. Black, red, and blue curves correspond to total SE, SE1, and SE2, respectively, and the ratio is taken for each respective signal.

To understand how the signal is modulated by the structure, we have calculated the lateral distributions of SE, SE1 and SE2 for electron beam landing at the different positions, i.e. the sphere top (particle diameter ∼10 nm), inner side of sphere edge (at a distance of −0.1 nm away), outer side of sphere edge (at a distance of 0.4 nm away) where the 0.4 nm position corresponds to the center of a gap between two gold particles for the resolution measurement of 0.8 nm, respectively. Then the ratio of lateral distributions between the particle/substrate case and planar case (Figure 8) is taken and shown in Figure 15 for 10 and 0.75 keV. In 10 keV case, Figure 15a indicates that the secondary electron emission is greatly enhanced for the beam landing at the particle mainly from SE2, which is certainly related to the increased emission probability (or increased effective emission surface area) from the particle edge as compared with the planar surface. When an electron beam is incident on the particle edge, the secondary electron emission is suppressed in the nearby particle side, as illustrated in the right half region of particle occupied space in Figure 15b. Figure 15c shows that the suppression in the particle region holds for an electron beam landing at a short distance away from the particle edge. This may be metaphorically described as the attenuation of SE2 emission by an additional absorber, i.e., the particle. Be reminded that the SE2 are not produced directly by the backscattered electrons during their penetration of the surface layer of ∼1 nm thick, but are mostly produced in the interior of the bulk (the first generation), and only those cascade generations of low energies can have a certain probability to escape into the vacuum (Figure 4). An additional surface layer, even in several nanometers thick would likely reduce the kinetic energy of secondary electrons during transportation and lowers down their emission intensity. This blocking effect functions like a screening of electron emission by a miniature Faraday cup, when an electron beam falls into a particle gap where the surrounding particles play as the cup wall to reduce the distant emission.

At low energies below 1 keV (Figure 15d‐‐f) the primary electrons become hard to reach the substrate by penetrating through the gold particle. In an in‐lens SEM system, the strong extraction field efficiently collects nearly all emitted secondary electrons without allowing them to re‐enter the nearby structure surface, and the miniature Faraday cup is still effective even at sub‐keV energies (Figure 15f). Even in the left half region of the particle occupied space the SE2 emission intensity ratio is seemly enhanced greatly in Figure 15f as compared with Figure 15c, but the distant SE2 absolute emission intensity is also greatly reduced at low energies (Figure 8f) and the emission range is reduced more than one order of magnitude as compared with high energy (Figure 10b). While the quantitative magnitude of SE2 suppression depends on beam energy, the morphology‐induced modulation of SE2 should remain significant across the sub‐keV region.

This miniature Faraday cup effect is not limited to the nanoparticles system studied here; it should be generally more or less applicable to other structured surfaces, including rough or textured surfaces, particularly if a sharp gap‐like concave region exists. When an electron beam enters such a concave region, the surrounding morphology behaves like a miniature Faraday cup and suppresses the distant SE2 emission. Conversely, when the beam impacts on the convex region, the morphology allows increased escape probability of SE1 and SE2 to enhance the image contrast between the concave and convex regions for features, such as, grooves, pores or rough surfaces.

To quantify the miniature Faraday cup effect, we compare the line‐scan profile over the particle with the substrate‐only yield, as shown in the bottom row of Figure 14. The deviation of each component from unity directly indicates the magnitude to which the electron yield is enhanced (values > 1) or suppressed (values < 1) at different beam positions. Figure 14f clearly shows that the SE2 signal is suppressed near the particle edge, with the suppression becoming more pronounced at lower beam energies. This confirms that the surrounding particle structure acts as an effective barrier, reducing SE2 emission in the gap region. It should be noted that the exact line‐scan profile depends on both the sample morphology and the material properties, and, therefore, the quantitative magnitude of the effect varies across different spatial regions (for a practical Au particle/C substrate sample, the SE2 emission in a carbon gap region is more localized as seen in Figures 5 and 6). The present simulations consistently show that the particle geometry directly modulates the spatial distribution of SE2, thereby enhancing image contrast at particle edges rather than merely contributing an undesirable background signal.

### 3.7 Edge Sharpness and Resolution

We now verify quantitatively that with SE2 the sub‐nanometer resolution is achievable. Regarding to the SEM image resolution, although there are several definitions reported so far in literature [75] it has still not been defined in scientifically sound ways according to an ISO document [76]. It is mentioned that “the notion of resolution is not established scientifically, it is sample‐ and method‐dependent, and there is no accurate way of measuring it today” [76]. Historically, the Rayleigh criterion defines the resolution of an optical instrument as the separation of two adjacent light spots distinguishable in an image. Following the principle of the Rayleigh criterion, the present ISO definition of SEM image resolution is given as the “minimum spacing at which two features of the image can be recognized as distinct and separate” [77]. This edge‐to‐edge resolution [78] was employed by some SEM manufacturers decades ago. Partly due to its difficulty of practical operation (e.g., the sample preparation and human sense for the minimum separation of particles), presently the edge sharpness is widely used as a replacement of Rayleigh criterion for SEM image resolution. Nevertheless, the edge sharpness still has several different metrics.

Assuming that the signal intensity profile *I*_0_(*x*) of a particle in in the shape of step function Θ(*x*), similar to that shown by Figure 14a for an ideal incident beam with infinitesimal probe size (*d_p_* = 0), and that the electron beam profile can be described by a normal distribution*N*(0, σ^2^), i.e. Gaussian function with standard deviation σ,

(14) $$G\left(x\right)=\frac{1}{\sqrt{2\pi \sigma^{2}}}\exp\left(-\frac{x^{2}}{2\sigma^{2}}\right)$$

where the beam diameter or probe size *d_p_* is given by the FWHM as [79],

(15) $$d_{p}=2\sqrt{2\ln2}\sigma \approx 2.355\sigma$$

**FIGURE 16:**
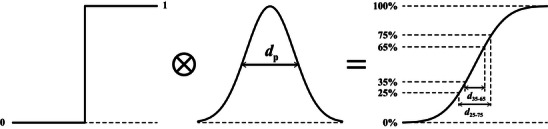
Schematic diagram of edge profile formation and sharpness metrics.

Then the edge intensity profile *I*(*x*) measured with this electron beam of finite probe size is a convolution of intensity profile *I*_0_(*x*) with the beam profile *G*(*x*). The result is in the shape of error function,

(16) $$\mathrm{erf}\left(x\right)=\Theta \left(x\right)⊗G\left(x\right)$$

as schematically shown by Figure 16. The sharpness of an edge profile *I*(*x*), which is normalized to be in the range of [0,^1^], is then given by the distance, *d*_α − β_, between two specified threshold values, (α%, β%), of *I*(*x*). For example, *d*_25 − 75_ corresponds to thresholds of (0.25, 0.75), which is greater than *d*_35 − 65_. These two sharpness definitions, *d*_25 − 75_ and *d*_35 − 65_, are often employed by different SEM manufacturers in presenting their resolution figures. On the other hand, there is an ISO definition of sharpness as $\sqrt{2}\sigma$ when *I*(*x*) is fitted to an error function of standard deviation σ [80], which is effectively *d*_24 − 76_, corresponding to thresholds of (0.24, 0.76).

However, the intensity profile of a particle *I*_0_(*x*) is actually not a simple step function and the profile shape depends on the size and shape (and also material) of a particle as well as primary energy. The edge intensity profile *I*(*x*) can be approximated as, in analogous to Equation (16),

(17) $$I\left(x\right)=I_{0}\left(x\right)⊗G\left(x\right)$$

**FIGURE 17:**
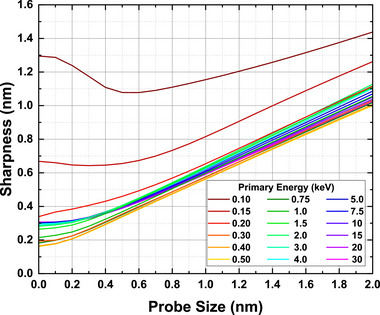
The evaluated sharpness *d*_25 − 75_ values as functions of probe size *d_p_* at the gold particle edge in Figure 14a. Colors indicate beam energy: 0.1–0.5 keV (dark red to yellow), 0.75–4 keV (dark green to cyan), and 5–30 keV (dark blue to magenta).

For the present example case of 10 nm gold sphere on a gold substrate, by using the calculated *I*_0_(*x*) curves presented in Figure 14a we have obtained the convoluted intensity profile *I*(*x*) for the electron beam with a finite probe size of *d_p_*, as shown in Figures S9. According to the sharpness metrics we have determined the “resolution”, *d*_25 − 75_, values as functions of *d_p_*, as shown in Figure 17. The figure clearly indicates that, with SE2 included as imaging signals, for a 10–30 keV beam with a probe size of *d_p_* ≈ 1 nm, the “resolution” value of $d_{25-75}∼0.6$ nm is attainable. As mentioned previously, the considered Au/Au sample geometry is more severe for resolution estimation. When a practical Au/C sample is considered, the lower secondary electron emission from carbon than gold leads to an even sharper edge profile and, hence, a smaller value of *d*_25 − 75_.

Therefore, we find in this work that so long as the edge sharpness is taken as image resolution, the sub‐nanometer resolution is realizable with both SE1 and SE2 signals. It implies that, therefore, a sub‐nanometer resolution is available even with a larger probe size than 1 nm. The critical factor for sub‐nanometer resolution is, in addition to the field emission cathode, the in‐lens detector in the immersion lens system, which helps to efficiently collect all secondary electron signals, i.e., SE1 and SE2, emitted from the sample and to effectively remove strayed electrons, i.e., SE3 [7]. SE3 are produced inside the instrument chamber by backscattered electrons hitting on the surfaces of instrument components, e.g. objective lens pole piece and specimen chamber, and carry the lower resolution characteristics of backscattered electrons. In comparison, it is hard to achieve such a sub‐nanometer resolution with an ordinary Everhart‐Thornley detector, which is located below the electron‐optical column and collects most of the SE1, SE2, SE3, and some of the backscattered electrons as imaging signals.

**FIGURE 18:**
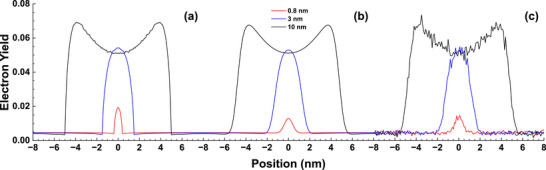
The line‐scan profiles for various diameters of a gold particle on a graphite substrate at a primary energy of 30 keV. (a) for an infinitesimal probe size, (b) the convoluted profile for a probe size of 0.8 nm, (c) Gaussian noise with $0.01\sqrt{\delta }$ standard deviation was added on the convoluted profile in (b), where δ is the secondary electron yield. Black, blue, and red curves correspond to particle diameters of 10, 3, and 0.8 nm, respectively.

Finally, in Figure 18, we show that even in the case of an individual gold particle on a carbon substrate for a practical resolution sample, for which the full miniature Faraday cup effect does not exist, the small nano‐sized particle is still visible. The 30 keV primary electrons are incident vertically onto the sample substrate plane and scan along the line passing through the sphere center. Figure 18a is the calculated line‐scan for an infinitesimal probe size and for three different sphere diameters. Figure 18b shows the convoluted line‐scans for a probe size of 0.8 nm. We further added some statistical noise, which is proportional to the square root of the signal intensity, as shown in Figure 18c. One can see that the sub‐nanometer particle can still be visible with the signal intensity distinct with the background noise according to the Rose criteria. The reason for this visibility is due to, according to Figure 15a, the SE1 from the gold particle is enhanced by the particle morphology, while the SE emission from carbon is rather weak as compared with gold. In a previous work [81] a Monte Carlo simulated SEM images of a pair of nanoparticles of an Au/C sample at 1 keV has been provided.

### 3.8 Limitation and Further Discussion

The present Monte Carlo simulations are based on several simplifying assumptions that should be noted when interpreting the results. The present model considers only two sphere particles in the same size with idealized smooth surfaces. This controlled condition allows us to reveal the intrinsic behaviors of SE1 and SE2 and to compare the results more directly with the planar surface simulations. Although real samples often possess more complex topography, typically rough surfaces with irregular shape, such rough surfaces can be decomposed into local convex and concave regions that resemble the particle/substrate geometry, where the local geometry effectively plays a role as if electrons are tilted incident, or impact closer or farer to a sphere edge. It is reasonable to expect the miniature Faraday cup effect still holds in such cases. Furthermore, in a practical resolution sample, the particle size may have a wide distribution. In case a tiny particle locates very close to a big one, the SE2 emission from the big particle by backscattered electrons emitted from the gap or tiny particle where the primary beam is incident could be significant, particularly at low beam energies, because of a much larger difference in particle heights. Then the contrast of the gap and tiny particle becomes faint, and the resolution is reduced by SE2.

The present simulation model also neglects charging effects in insulating materials. Surface charging can significantly alter the local electric field and subsequently modify secondary‐electron escape probabilities. While the charging phenomenon is beyond the scope of the present work, an advanced version of the Monte Carlo framework [45] could be incorporated in the future to extend the present insight about the role of SE2 in imaging of insulating samples.

It is useful to place the present SEM‐based analysis in the broader context of high‐resolution imaging techniques. Atomic force microscopy (AFM), transmission electron microscopy (TEM), and scanning transmission electron microscopy (STEM) routinely achieve sub‐nanometer down to atomic‐scale resolution, but their contrast mechanisms are fundamentally different from those of conventional SEM. TEM relies on electron transmission through thin specimens and therefore cannot be applied to bulk or thick samples, while AFM relies on mechanical probe–surface interactions and does not involve secondary electron emission. More than a decade ago, the atomic resolution secondary electron image has been achieved with an aberration corrected TEM operated in STEM mode (probe diameter ∼0.08 nm) at 200 keV for isolated uranium atoms on thin (2 nm) carbon films [82]. An incident electron beam at such high energy can fully penetrate through the ultrathin film without a chance to generate SE2; hence, the spatial resolution is determined by the probe size and SE1 cascade diffusion, where the first‐generation of secondary electron is resulted from the local atomic inner‐shell ionization whose binding energy is slightly lower than the beam energy [83]. Therefore, the imaging mechanism of this SEM/STEM type is also different from that of the conventional SEM discussed here, which is performed at much lower energy and for a bulk sample. For such geometries, the spatial distribution of SE1 and SE2—especially the morphology‐ modulate SE2 demonstrated here—directly influences the achievable resolution.

Beyond particle/substrate systems, the proposed secondary electron generation and transport framework is directly applicable to a broad range of emerging nanomaterials. For 2D materials it is interesting to investigate the contrast of the point defects and 2D material/substrate where the atomic thicknesses of the 2D materials may play a role for the miniature Faraday cup effect of SE2 emission. In this case, a modification of the present Monte Carlo model—particularly for the treatment of surface excitations [57] and the accompanied low‐energy secondary electron generations [84]—should be adapted to the imaging of a few layer systems. Similarly, quantum dots and other nanoscale semiconductor structures rely on local variations in surface potential, dielectric confinement, and band‐edge structure, all of which influence secondary electron yield and contrast at low beam energies. Because our method incorporates material‐specific properties, it can be extended to these systems by substituting their corresponding physical constants. Thus, while this study focuses on particle/substrate systems, the underlying modeling strategy is general and can support future investigations of 2D materials, quantum dots, and other emerging nanomaterial platforms.

For applications of SEM in materials science and semiconductor inspection, this insight suggests that enhanced resolution can be achieved through suppressing unwanted stray electrons (SE3) in addition to the reduction of probe size and signal noise, improved beam stability and intensity, rather than selective detection of SE1 and SE2. In biological imaging, where low‐dose and surface‐sensitive conditions are critical, an understanding of SE2 at charging conditions can also contribute meaningful support to the development of gentler imaging modes without compromising resolution.

## 4 Conclusion

In this work, to clarify the mechanism for ultrahigh resolution SEM imaging achieved by the immersion lens type SEM instruments, we have performed comprehensive Monte Carlo simulations of emission properties of secondary electron signals. With three different definitions of SE1 and SE2 we have found that these two types of electrons have very similar emission properties except that the emission radial distribution of SE2 is much wider than that of SE1. This disproves the hypothesis that the high‐resolution SE1 signals are more efficiently detected than the SE2. On the contrary, our simulations suggest that SE2 are important signals for ultrahigh resolution imaging of the resolution test sample made of nanosized gold particles. This is because, on the one hand, the population of SE2 is much greater than that of SE1, and on the other hand, 3D nanostructure in moderate size can modulate the spatial emission of SE2 to be more localized in the protruding structure region. The SE2 from a nanosized gap between two particles in resolution measurement is largely suppressed by the nearby particles, which is named as miniature Faraday cup effect. According to a method for resolution determination of SEM image, the edge sharpness of the intensity profile with both SE1 and SE2 counted as signals around a particle edge is evaluated as a function of probe size. The results show that theoretically, the sub‐nanometer resolution is attainable, although the beam aberration and finite probe size in practical systems may influence the attainable resolution. Overall, the key factors enabling ultrahigh‐resolution SEM imaging are the effective exclusion of SE3 by the in‐lens detector and the morphology‐modulated emission of SE2.

## 5 Monte Carlo Model

The up‐to‐date Monte Carlo simulation model with the latest electron elastic and inelastic scattering cross sections is utilized in the present calculation. Details of this procedure are described elsewhere [32, 39], and an extended version of this model is capable of handling arbitrary 3D geometries and has been validated in previous studies [53, 55]. For clarity, we provide only a concise overview of the calculation steps here.

### 5.1 Electron Elastic Scattering

The Mott's differential cross section [51] by solving the Dirac equation describes accurately the elastic scattering of electrons by atoms in a solid, particularly at very low energies [31],

(18) $$\frac{d\sigma_{e}}{d\Omega }={\left|f\left(\theta \right)\right|}^{2}+{\left|g\left(\theta \right)\right|}^{2}$$

where *f*(θ) and *g*(θ) are the scattering amplitudes and can be calculated with the partial wave expansion method [85]. In this work, the atomic scattering potential contains three parts, i.e., the electrostatic potential, the exchange potential, and the correlation‐polarization potential. The Fermi distribution and the Dirac–Fock electron density are used to determine the nuclear and electronic charge‐density, respectively [86]. In addition, the Furness–McCarthy exchange potential, and the correlation‐polarization potential [87] based on the local‐density‐approximation [88] are also considered. The Mott's cross section is calculated with the ELSEPA program [85].

### 5.2 Electron Inelastic Scattering

The dielectric functional formalism is used to determine the electron inelastic scattering cross section, which is inversely proportional to the electron IMFP. In this model, the differential inverse IMFP (DIIMFP) for moving electrons in a material is written as:

(19) d2λin−1dℏωdq=2γ21+γ1πa0EIm−1εq,ω1q

where $\lambda_{in}$ is the IMFP. ε(*q*, ω) is the complex dielectric function of a medium. The probability of the inelastic scattering events is determined by the energy loss function (ELF), Im{−1/−1ε(q,ω)ε(q,ω)}. Penn has suggested an algorithm [50], for the extension of the optical ELF, Im{−1/−1ε(0,ω)ε(0,ω)}, whose data are experimentally available, from the optical limit of *q*  → 0 into the  (*q*, ω)‐plane. However, it should be noted that the low‐energy electron inelastic scattering is inherently a multi‐electron quantum process, where the exchange‐correlation effect omitted here may not be a minor correction. Meanwhile, the secondary electron generation nearby the surface via the decay of surface plasmon is omitted.

**FIGURE 19:**
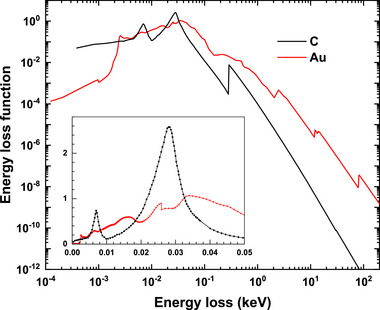
ELFs of C (graphite, black) and Au (red) used in this work.

To check the accuracy of the optical ELF data used, one can apply the oscillator strength sum rule (*f*‐sum rule) and the perfect screening sum rule (*ps*‐sum rule) [89]. It has been indicated that the accuracy of ELF data evaluated through the error of sum‐rules influences the Monte Carlo simulated electron emission yields [90]. In this work, the optical ELF data of the concerned elemental solids are taken from a database of optical constants and are the same as that used for the calculation of IMFP [91]. Figure 19 shows the plots of optical ELFs as functions of energy loss or the photon energy ℏω. The corresponding *f*‐sum rules and *ps*‐sum rules are provided in Table 3.

**TABLE 3:** The *f*‐ and *ps*‐sum rules and their relative errors for C (graphite) and Au.

	*f*‐sum rule		*ps*‐sum rule	
	value	relative error (%)	value	relative error (%)
C	6.29	4.8	1.077	7.7
Au	75.99	−1.8	1.089	8.9

### 5.3 Electron Transportation

When a kinetic electron is incident into a sample, it will undergo elastic and inelastic collisions, so the direction of movement and the kinetic energy will be changed. The scattering angle and the energy loss can be sampled by the respective differential cross sections with random numbers in a Monte Carlo simulation procedure. If the lost energy ℏω satisfies ℏω  < *E_B_*, where *E_B_* is the smallest binding energy of the observable ionization edge of inn‐shells presented in the plot of the optical ELF, then a secondary electron is assumed to be excited from the Fermi sea by transferring the energy ℏω from the kinetic electron to a valence electron, with the excitation probability being proportional to a joint density of states of free electrons. Otherwise (ℏω  > *E_B_*), a secondary electron is excited from an inner shell of the atom [92]. In addition, the relaxation of excited atoms may proceed via the emission of an Auger electron or a photon. However, the contribution of Auger electrons to the emitted electron signals is negligible due to the low probability of inner‐shell ionization.

The excited secondary electrons are further tracked through similar elastic and inelastic scattering processes as a primary electron. During this transport, they can generate tertiary and higher‐order secondary electrons, which we collectively refer to as cascade secondary electrons. In this cascade process, a single incident electron can therefore produce a large number of secondary electrons within the solid [33].

### 5.4 Electron Emission

After undergoing multiple scattering events inside the sample, an electron may reach the surface region. It can then escape from the sample with a certain probability, i.e., the transmission function *T*. In this work, a quantum mechanical transmission function [93] is used,

(20) TE,β=41−U0U0Ecos2βEcos2β1+1−U0U0Ecos2βEcos2β2,ifEcos2β>U0;0,otherwise,

where β is the angle between the electron moving direction and the surface normal, and *U*_0_ is the sum of the work function and Fermi energy for a conductor [40]. The work functions and Fermi energies of C and Au are provided by Table 4. According to the kinetic energy referenced to the vacuum level, an escaped electron is counted either as a true secondary electron signal (<50 eV) or a backscattered electron signal (>50 eV).

**TABLE 4:** The work function and Fermi energy values for C (graphite) and Au [94].

	Work function (eV)	Fermi energy (eV)
C	5.0	20.4
Au	5.1	5.53

## Author Contributions

H.T.C. conducted the formal analysis and investigation, developed the methodology, and prepared the original draft of the manuscript. B.D. carried out the investigation and acquired funding. Y.B.Z. contributed to the investigation and secured funding. Z.J.D. was responsible for conceptualization, formal analysis, investigation, and methodology, and oversaw project administration and supervision, as well as reviewing and editing the manuscript.

## Conflicts of Interest

The authors declare no conflicts of interest.

## Supporting information




**Supporting File 1**: advs73849‐sup‐0001‐SuppMat.docx.

**Supporting File 2**: advs73849‐sup‐0002‐FigureS1‐S11.zip.

## Data Availability

The data that support the findings of this study are available from the corresponding author upon reasonable request.
