# Supplementary material for: A Quest for the Mechanism of Ultrahigh Resolution SEM Imaging
Source: Adv Sci (Weinh). 2026 Jan 26;13(17):e16341. doi: 10.1002/advs.202516341 (PMC13042853; doi:10.1002/advs.202516341)
Supplement: Supplementary file 1 — Supporting File 1: advs73849‐sup‐0001‐SuppMat.docx. [file ADVS-13-e16341-s002.docx]

**Supplementary Information for A Quest for the Mechanism of Ultrahigh Resolution SEM Imaging**

Haotian Chen^1^, Y. B. Zou^2#^, B. Da^3§^ and Z. J. Ding^1*^

*^1^Hefei National Research Center for Physical Sciences at the Microscale and Department of Physics, University of Science and Technology of China, Hefei, Anhui 230026, P.R. China*

^2^*School of Physics & Electronic Engineering, Xinjiang Normal University, Urumqi, Xinjiang 830054, P.R. China*

^3^*Center for Basic Research on Materials, National Institute for Materials Science, Tsukuba, Ibaraki 305-0044, Japan*

^#^Corresponding author: [zouyb@xjnu.edu.cn](mailto:zouyb@xjnu.edu.cn)

^§^Corresponding author: DA.Bo@nims.go.jp

^*^Corresponding author: [zjding@ustc.edu.cn](mailto:zjding@ustc.edu.cn)

# Supplementary methods

The trajectory of each electron (primary or secondary) is traced by a standard event-by-event Monte Carlo scheme. The distance to the next scattering event is sampled from an exponential distribution with total mean free path

$\frac{1}{\lambda_{T}}=\frac{1}{\lambda_{e}}+\frac{1}{\lambda_{in}}$, (1)

where $\lambda_{e}$ and $\lambda_{in}$ are the elastic and inelastic mean free paths. A random number $R_{1}\in[0,1]$ gives the step length:

$s=-\lambda_{T}\ln R_{1}$. (2)

A second random number $R_{2}\in[0,1]$ determines whether the event is elastic or inelastic:

$\left\{\begin{matrix} \text{elastic}, & \text{if } R_{2}<\lambda_{e}^{-1}/\lambda_{T}^{-1}; \\ \text{inelastic}, & \text{otherwise.} \end{matrix}\right.$ (3)

If the event is elastic, the scattering angle is sampled from the Mott's elastic differential cross section with cumulative sampling, and the azimuthal angle is isotropic. If the event is inelastic, the energy loss is firstly sampled from the *q*-integrated DIIMFP,

(4)

by using cumulative sampling in the energy loss range of $[0,E-E_{F}]$, where $q_{\pm}$ are the upper and lower bounds of the integration [1], $E_{F}$ is the Fermi energy and *E* is the kinetic energy of moving electron. Then, a second sampling of momentum transfer *q* from DIIMFP, by setting energy loss to the sampled value, yields the corresponding scattering angle:

$\cos\theta=\frac{q_{+}^{2}+q_{-}^{2}-2q^{2}}{q_{+}^{2}-q_{-}^{2}}$. (5)

After the inelastic scattering, the electron energy becomes .

Whenever an inelastic scattering event occurs, the energy loss may result in a secondary electron generation. If the lost energy satisfies , where $E_{B}$ is the smallest binding energy of the observable ionization edge of inn-shells presented, then a secondary electron is assumed to be excited from Fermi sea by transferring the energy from the kinetic electron to a valence electron with the excitation probability being proportional to the joint density of states of free electrons [2], denoted as . Otherwise (), a secondary electron is excited from an inner shell of the atom with a probability distribution of the initial state is described by [3]:

, (6)

where **k**_i_ is the initial wave vector of the electron, **k**_F_ is the Fermi wave vector, and **k**_f_ = **k**_i_ +**q** is the wave vector of the excited secondary electron. The secondary electron is assumed to originate at the location of the inelastic event and its initial moving direction is taken as isotropic. Each generated secondary electron is then transported under the same Monte Carlo procedure as for the primary electron. Secondary electrons may generate again higher-order cascade secondary electrons. All such electrons are collectively treated as cascade secondary electrons and are followed until their escape or absorption.

# Supplementary results

Monte Carlo simulations were performed for 10^8^ primary electron trajectories for a planar surface. For a 30 keV incident beam, the simulation required approximately 20 hours on a 400-core workstation. The total runtime scales approximately linearly with beam energy. For example, a 10 keV simulation requires about 7 hours under the same condition.

For simulations on the particle/substrate system, the modeled surface is meshed via the finite-element method into 641 triangular surface elements. In this case, 10^6^ primary electron trajectories were traced, and the total computation time for a 30 keV beam is approximately 10 hours on the same 400-core workstation. The statistical fluctuation of Monte Carlo simulated secondary yields and line-scan profile is thus in the order of 10^-2^-10^-3^.

**Supplementary Figure 1** Weighted radial distributions for graphite. The left, middle and right columns are for SE, SE1 and SE2 components, respectively, given by Eq. (14). The top, middle and bottom rows are calculated by the definition-1, -2 and -3, respectively. Colors indicate beam energy: 0.1–0.5 keV (dark red to yellow), 0.75–4 keV (dark green to cyan), and 5–30 keV (dark blue to magenta).

**Supplementary Figure 2** Weighted radial distributions for gold. The left, middle and right columns are for SE, SE1 and SE2 components, respectively, given by Eq. (14). The top, middle and bottom rows are calculated by the definition-1, -2 and -3, respectively Colors indicate beam energy: 0.1–0.5 keV (dark red to yellow), 0.75–4 keV (dark green to cyan), and 5–30 keV (dark blue to magenta).

**Supplementary Figure 3** The ratio of SE1 and SE2 components in radial distribution of graphite. The left, middle and right columns are $I'_{\text{SE1}}\left(r\right)/I'_{\text{SE}}\left(r\right)$, $I'_{\text{SE2}}\left(r\right)/I'_{\text{SE}}\left(r\right)$ and $I'_{\text{SE1}}\left(r\right)/I'_{\text{SE2}}\left(r\right)$, respectively, where the top, middle and bottom rows are calculated by the definition-1, -2 and -3, respectively. Colors indicate beam energy: 0.1–0.5 keV (dark red to yellow), 0.75–4 keV (dark green to cyan), and 5–30 keV (dark blue to magenta).

**Supplementary Figure 4** The ratio of SE1 and SE2 components in radial distribution of gold. The left, middle and right columns are $I'_{\text{SE1}}\left(r\right)/I'_{\text{SE}}\left(r\right)$, $I'_{\text{SE2}}\left(r\right)/I'_{\text{SE}}\left(r\right)$ and $I'_{\text{SE1}}\left(r\right)/I'_{\text{SE2}}\left(r\right)$, respectively, where the top, middle and bottom rows are calculated by the definition-1, -2 and -3, respectively. Colors indicate beam energy: 0.1–0.5 keV (dark red to yellow), 0.75–4 keV (dark green to cyan), and 5–30 keV (dark blue to magenta).

**Supplementary Figure 5** The radial statistic metrics for graphite. The top, middle and bottom columns are the 68th percentiles $R$, the expectation values $\bar{r}$ of radial distributions, and the standard deviations $\sigma_{r}$ of radial distributions, respectively, where the left, middle and right rows are calculated by the definition-1, -2 and -3, respectively. Black, red, and blue curves correspond to total SE, SE1, and SE2, respectively.

**Supplementary Figure 6** The radial statistic metrics for gold. The top, middle and bottom columns are the 68th percentiles $R$, the expectation values $\bar{r}$ of radial distributions, and the standard deviations $\sigma_{r}$ of radial distributions, respectively, where the left, middle and right rows are calculated by the definition-1, -2 and -3, respectively. Black, red, and blue curves correspond to total SE, SE1, and SE2, respectively.

**Supplementary Figure 7** Weighted depth distributions for graphite. The left, middle and right columns are for SE, SE1 and SE2, respectively, where the top, middle and bottom rows are calculated by the definition-1, -2, and -3, respectively. Colors indicate beam energy: 0.1–0.5 keV (dark red to yellow), 0.75–4 keV (dark green to cyan), and 5–30 keV (dark blue to magenta).

**Supplementary Figure 8** Weighted depth distributions for gold. The left, middle and right columns are for SE, SE1 and SE2, respectively, where the top, middle and bottom rows are calculated by the definition-1, -2, and -3, respectively. Colors indicate beam energy: 0.1–0.5 keV (dark red to yellow), 0.75–4 keV (dark green to cyan), and 5–30 keV (dark blue to magenta).

**Supplementary Figure 9** Energy distributions for graphite. The left, middle and right columns are for SE, SE1 and SE2, respectively, where the top, middle and bottom rows are calculated by the definition-1, -2, and -3, respectively. Colors indicate beam energy: 0.1–0.5 keV (dark red to yellow), 0.75–4 keV (dark green to cyan), and 5–30 keV (dark blue to magenta).

**Supplementary Figure 10** Energy distributions for gold. The left, middle and right columns are for SE, SE1 and SE2, respectively, where the top, middle and bottom rows are calculated by the definition-1, -2, and -3, respectively. Colors indicate beam energy: 0.1–0.5 keV (dark red to yellow), 0.75–4 keV (dark green to cyan), and 5–30 keV (dark blue to magenta).

**Supplementary Figure 11** The convolution results of the line scans of Figure 18(a) with probe size $d_{p}$ of (a) 0.5 nm, (b) 1 nm, (c) 1.5 nm and (d) 2 nm. Colors indicate beam energy: 0.1–0.5 keV (dark red to yellow), 0.75–4 keV (dark green to cyan), and 5–30 keV (dark blue to magenta).

**Supplementary References**

[1] D. Pines, and P. Nozieres. *The Theory of Quantum Liquids: Normal Fermi Liquids* (Vol. 1). WA Benjamin. (1966).

[2] M. S. Chung, and T. E. Everhart. *Role of plasmon decay in secondary electron emission in the nearly-free-electron metals. Application to aluminum.* Physical Review B, *15*(10), (1977): 4699.

[3] J.P. Ganachaud and M. Cailler. *A Monte-Carlo calculation of the secondary electron emission of normal metals: I. The model.* Surface Science 83(2) (1979): 498-518.
