# Supplementary figures and images for: A Quest for the Mechanism of Ultrahigh Resolution SEM Imaging
Source: Adv Sci (Weinh). 2026 Jan 26;13(17):e16341. doi: 10.1002/advs.202516341 (PMC13042853; doi:10.1002/advs.202516341)

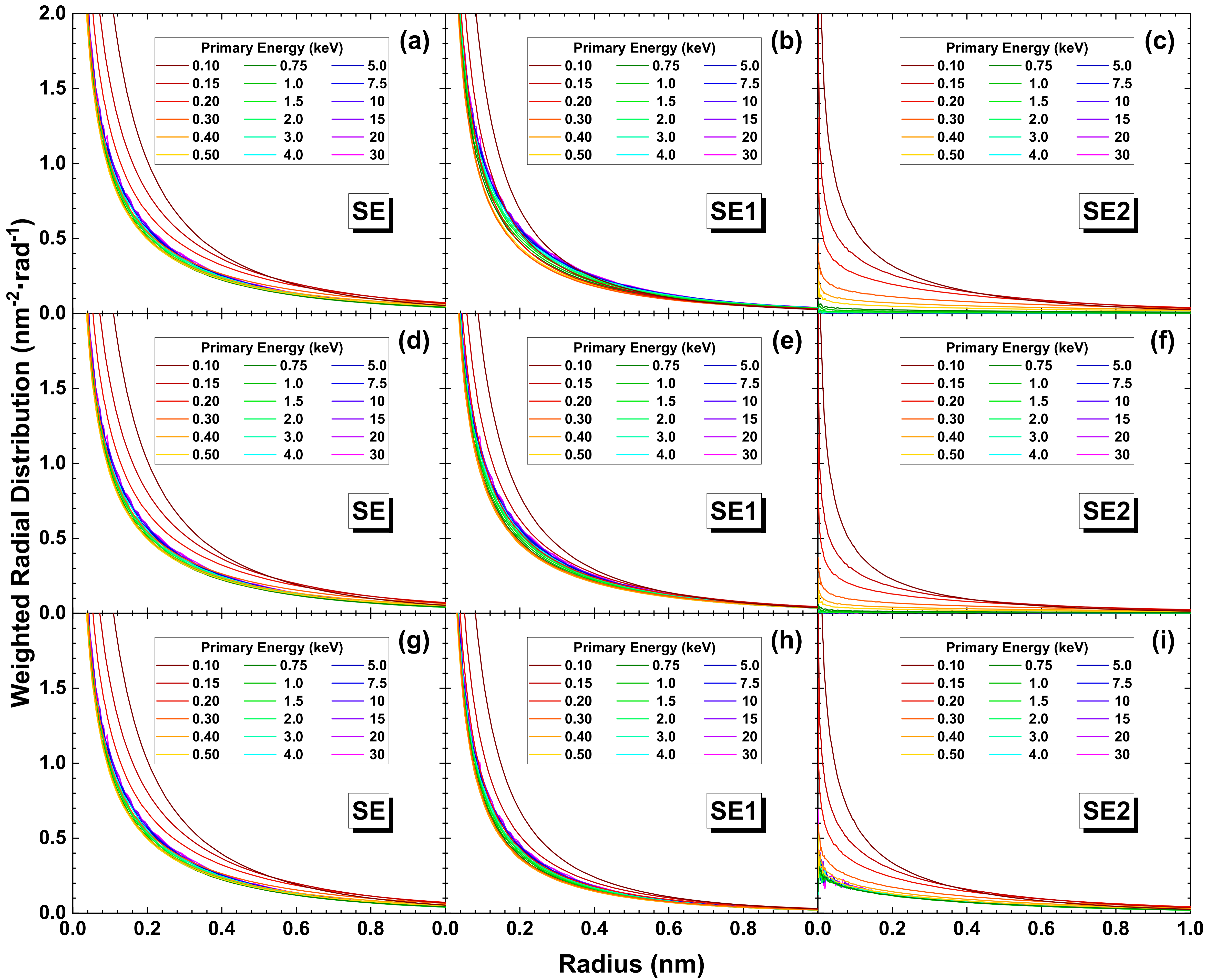

Supplement: Supplementary file 2 — Supporting File 2: advs73849‐sup‐0002‐FigureS1‐S11.zip. [file ADVS-13-e16341-s001.zip › Supplementary Figure 1.pdf]

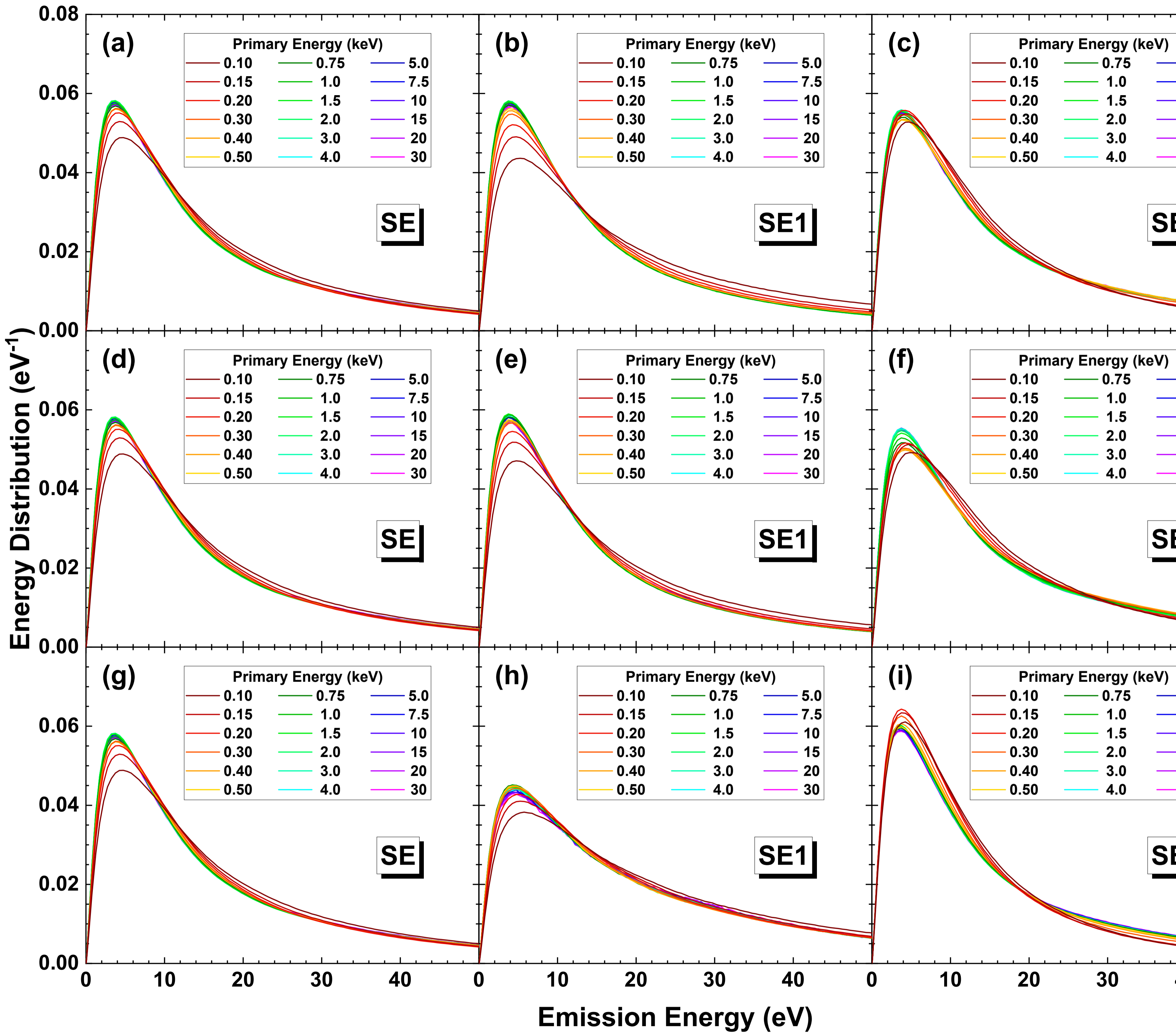

Supplement: Supplementary file 2 — Supporting File 2: advs73849‐sup‐0002‐FigureS1‐S11.zip. [file ADVS-13-e16341-s001.zip › Supplementary Figure 10.pdf]

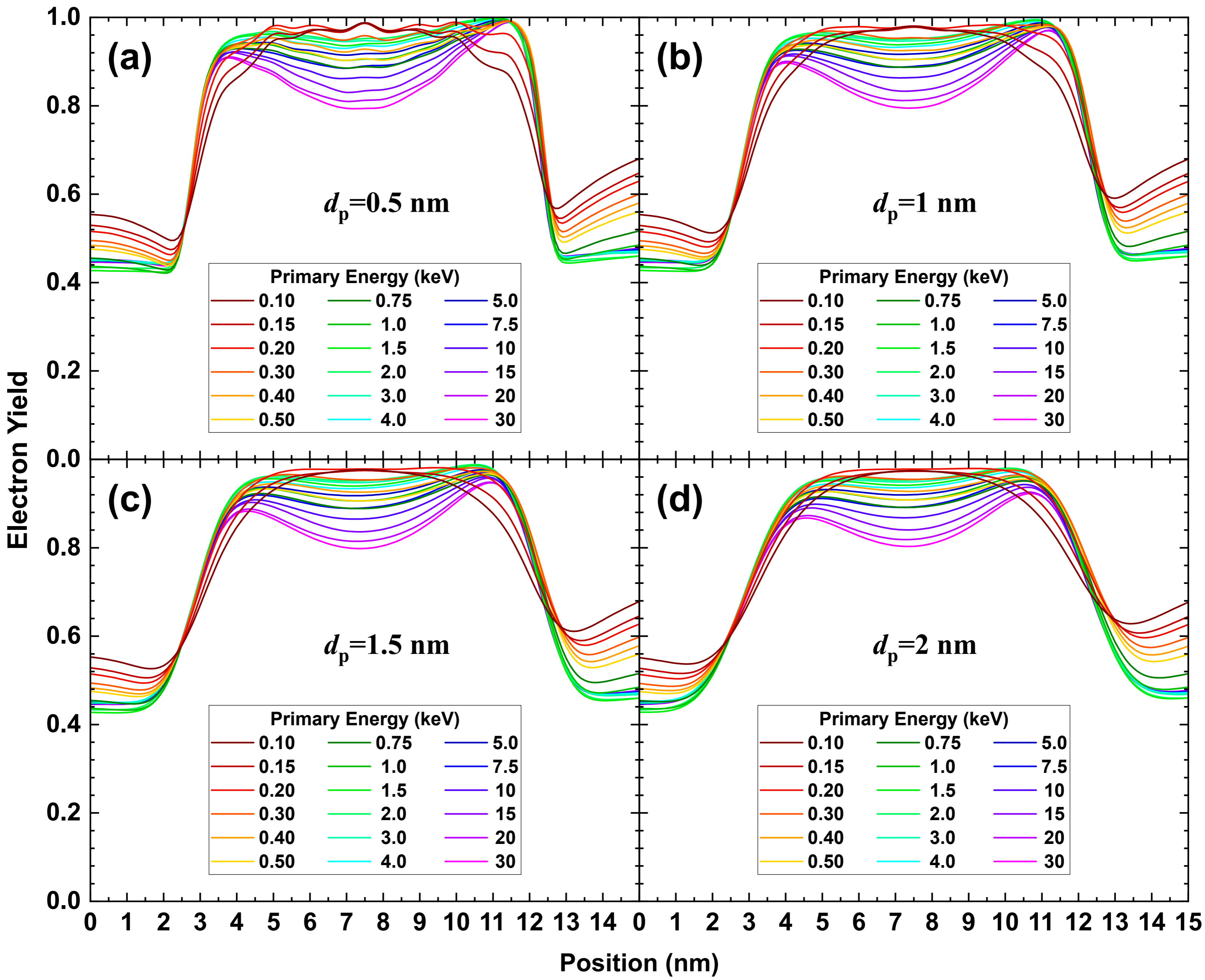

Supplement: Supplementary file 2 — Supporting File 2: advs73849‐sup‐0002‐FigureS1‐S11.zip. [file ADVS-13-e16341-s001.zip › Supplementary Figure 11.pdf]

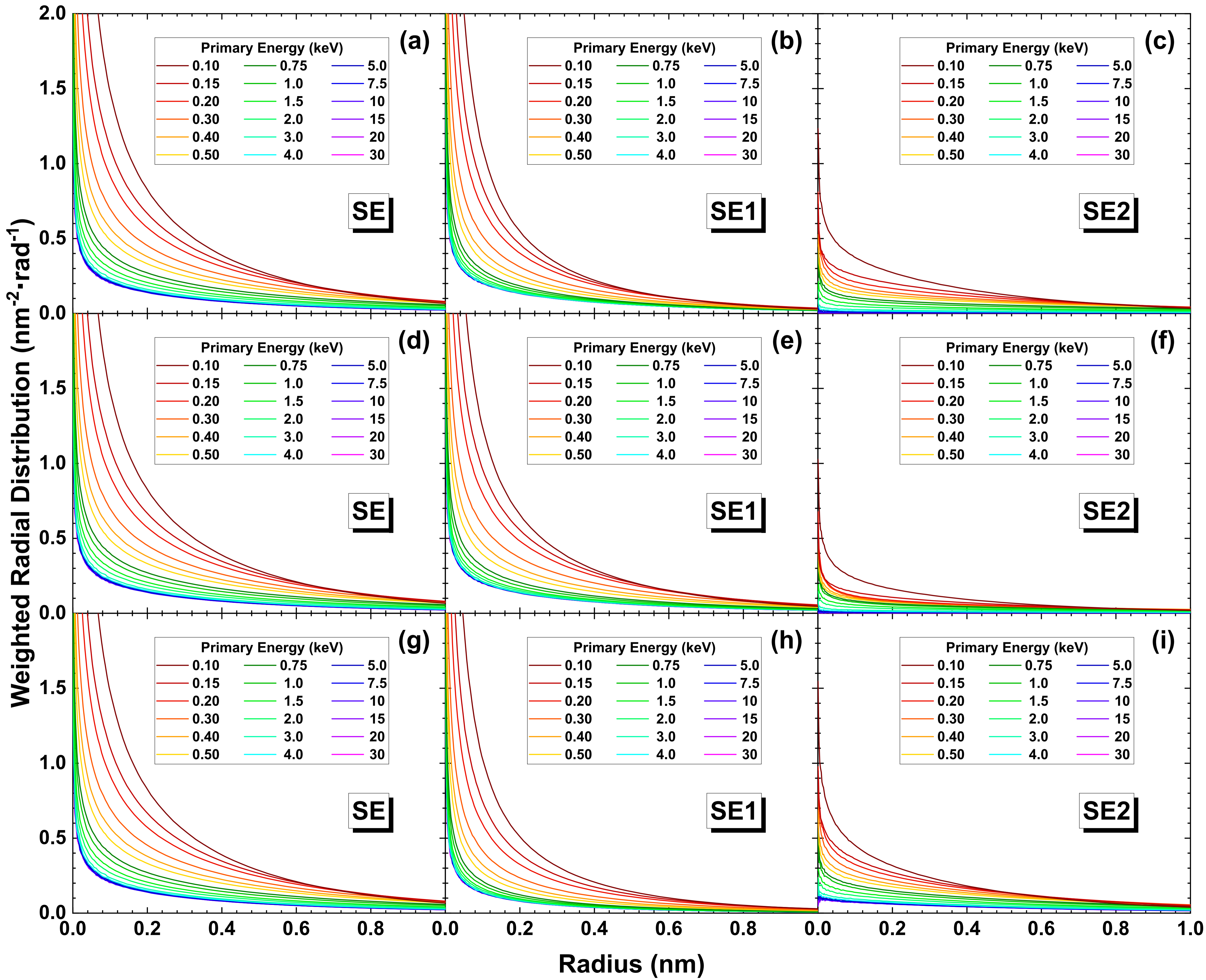

Supplement: Supplementary file 2 — Supporting File 2: advs73849‐sup‐0002‐FigureS1‐S11.zip. [file ADVS-13-e16341-s001.zip › Supplementary Figure 2.pdf]

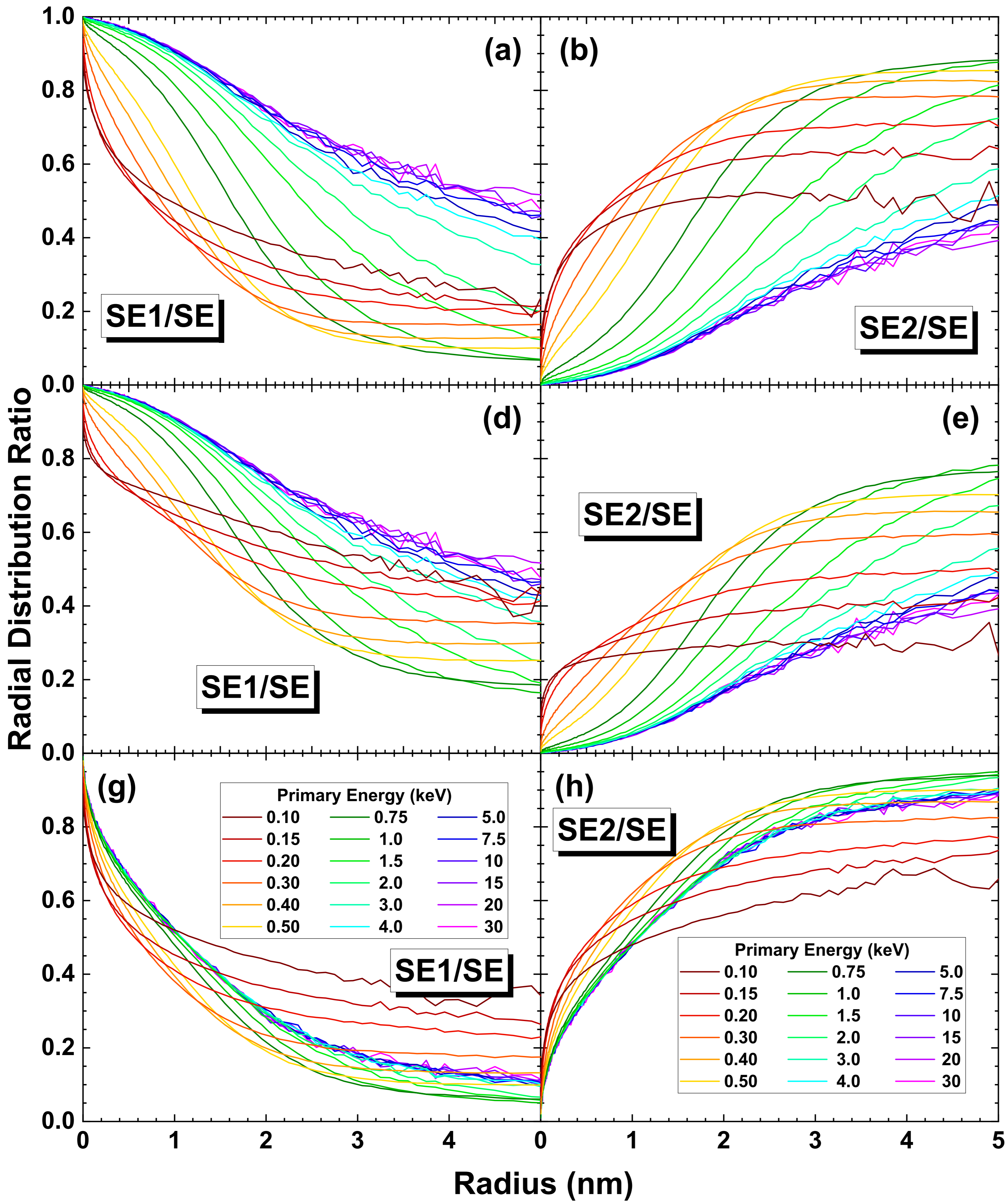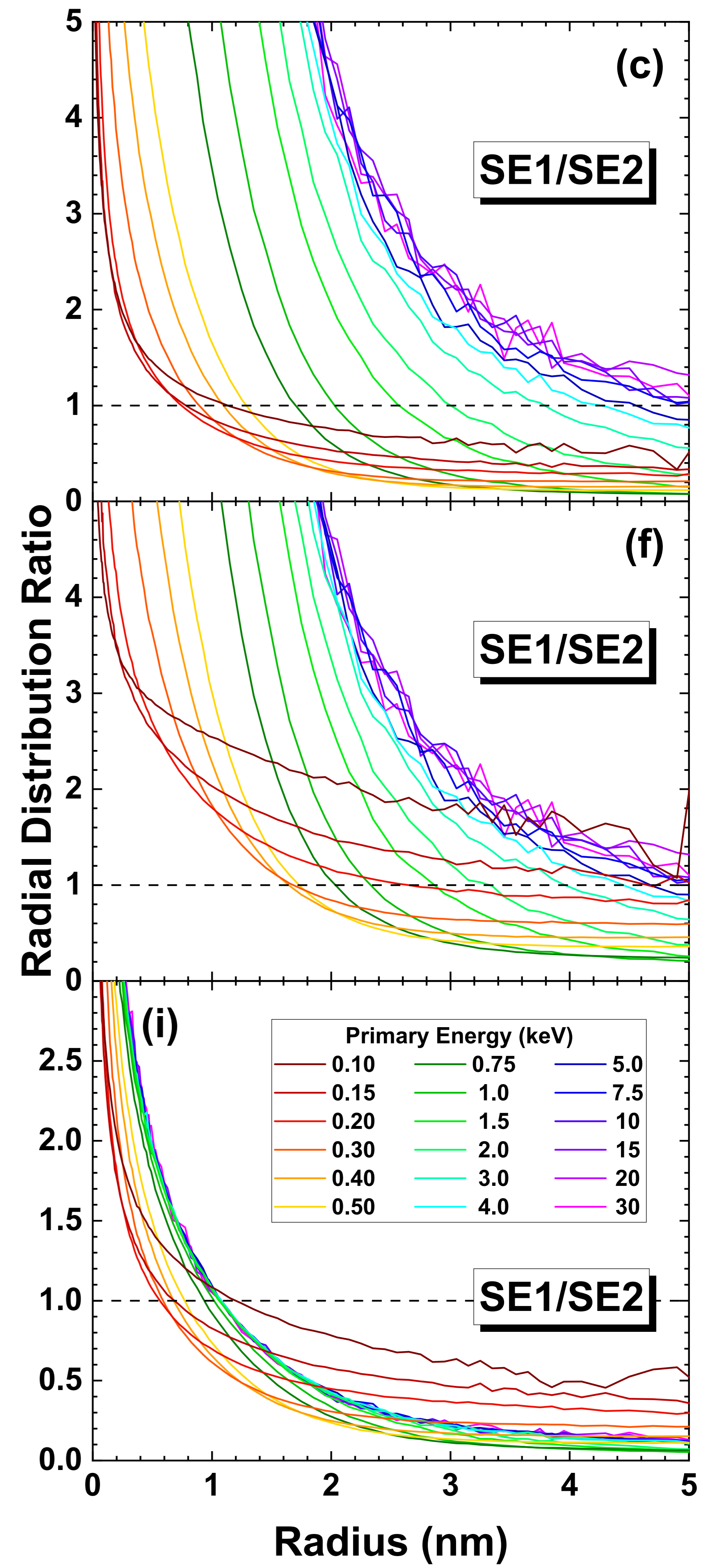

Supplement: Supplementary file 2 — Supporting File 2: advs73849‐sup‐0002‐FigureS1‐S11.zip. [file ADVS-13-e16341-s001.zip › Supplementary Figure 3.pdf]

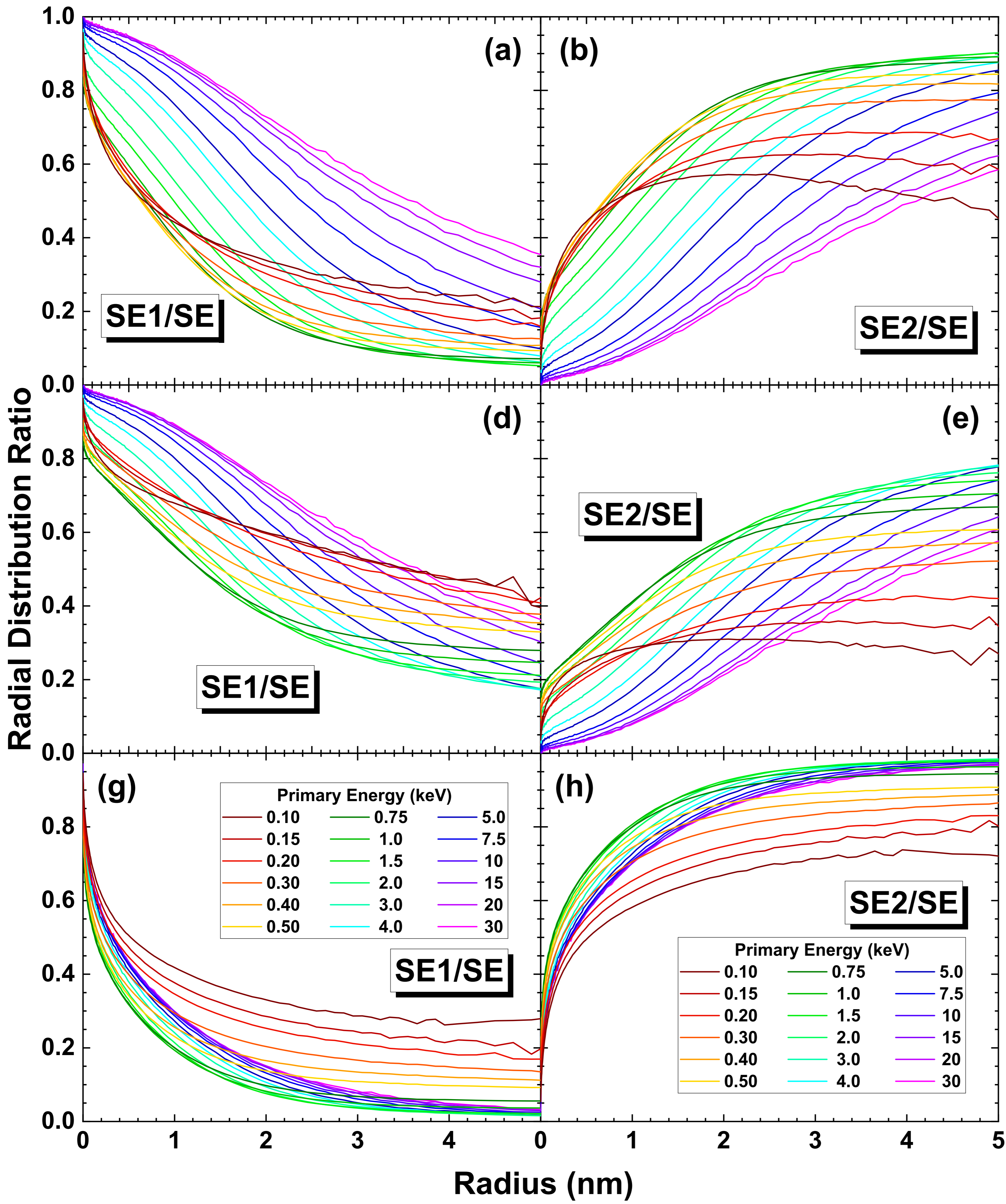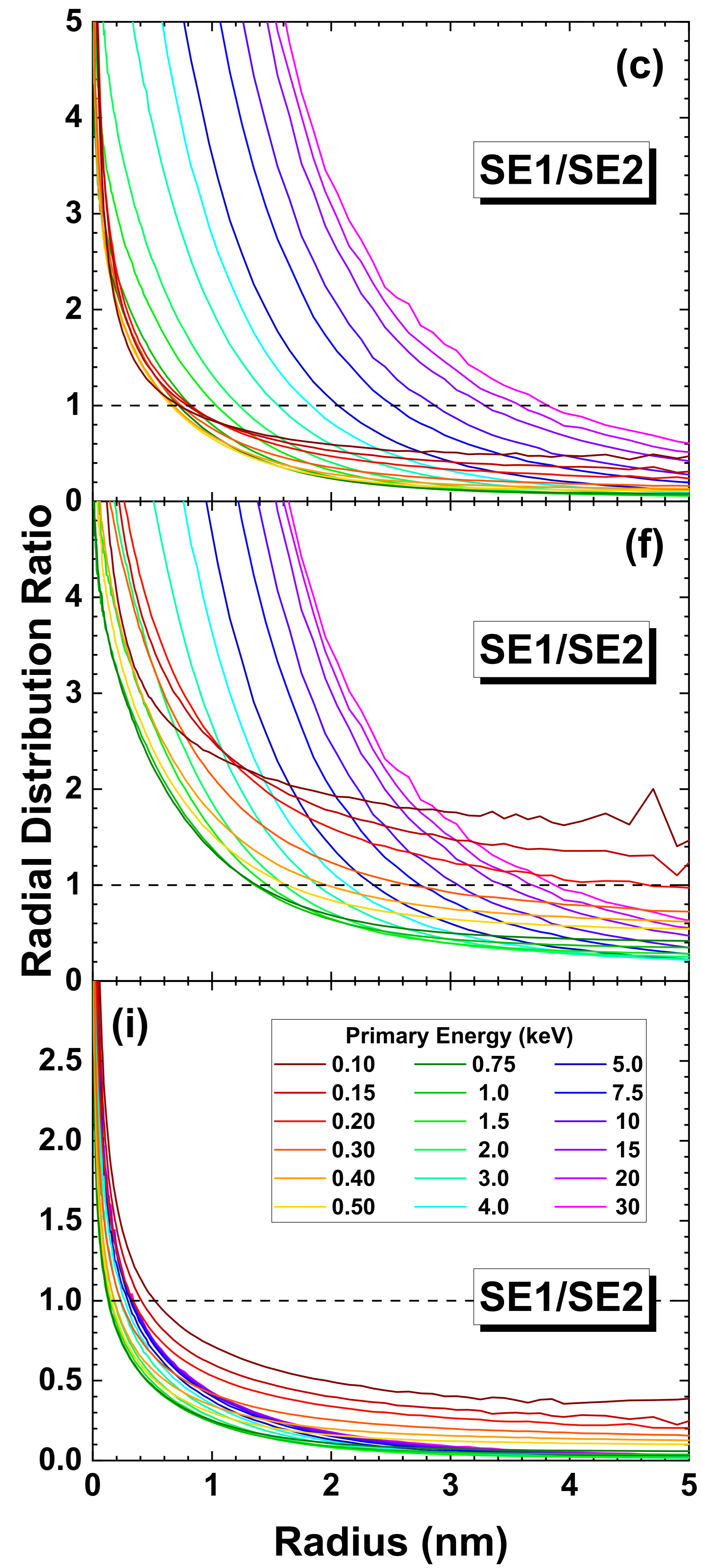

Supplement: Supplementary file 2 — Supporting File 2: advs73849‐sup‐0002‐FigureS1‐S11.zip. [file ADVS-13-e16341-s001.zip › Supplementary Figure 4.pdf]

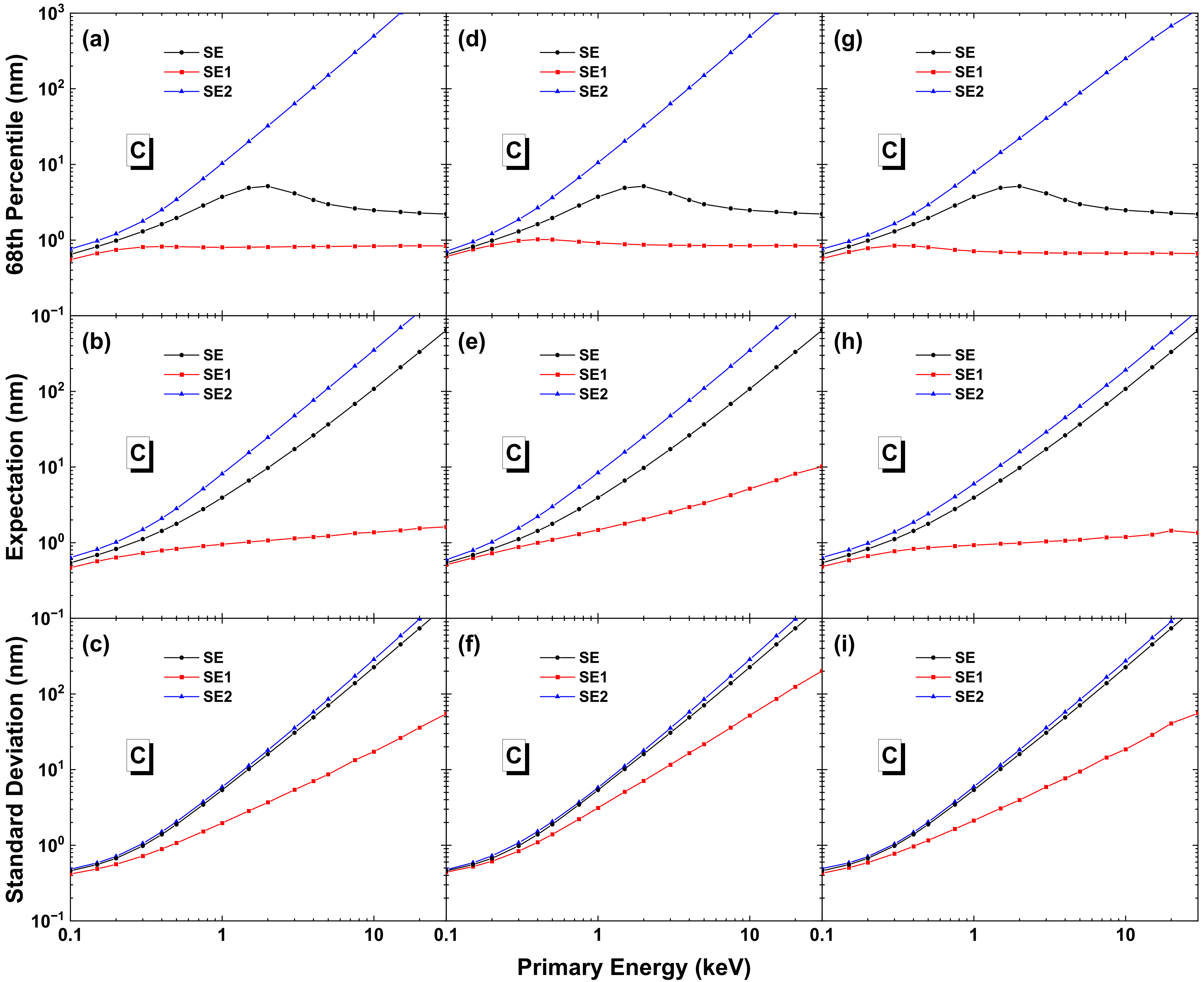

Supplement: Supplementary file 2 — Supporting File 2: advs73849‐sup‐0002‐FigureS1‐S11.zip. [file ADVS-13-e16341-s001.zip › Supplementary Figure 5.pdf]

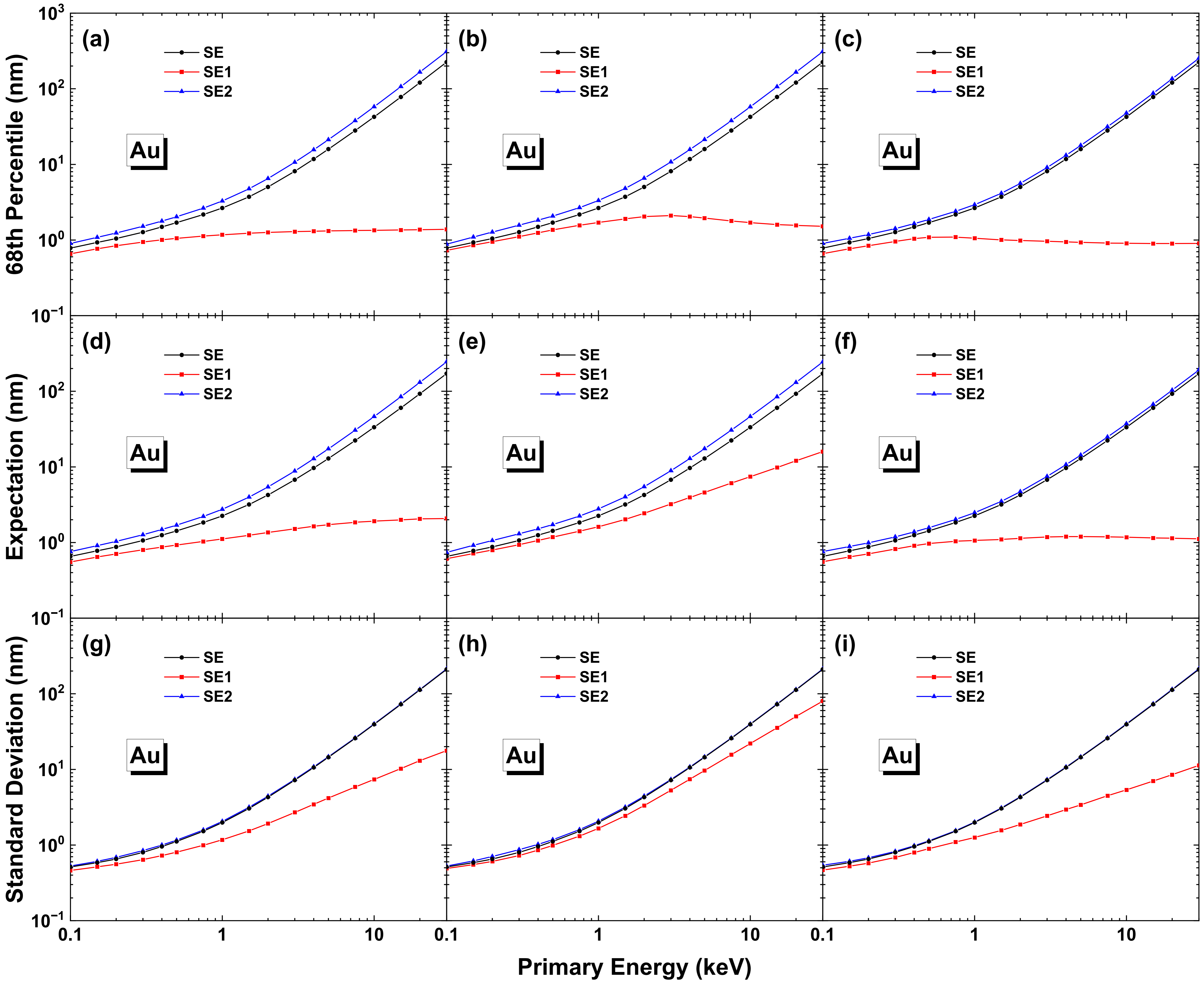

Supplement: Supplementary file 2 — Supporting File 2: advs73849‐sup‐0002‐FigureS1‐S11.zip. [file ADVS-13-e16341-s001.zip › Supplementary Figure 6.pdf]

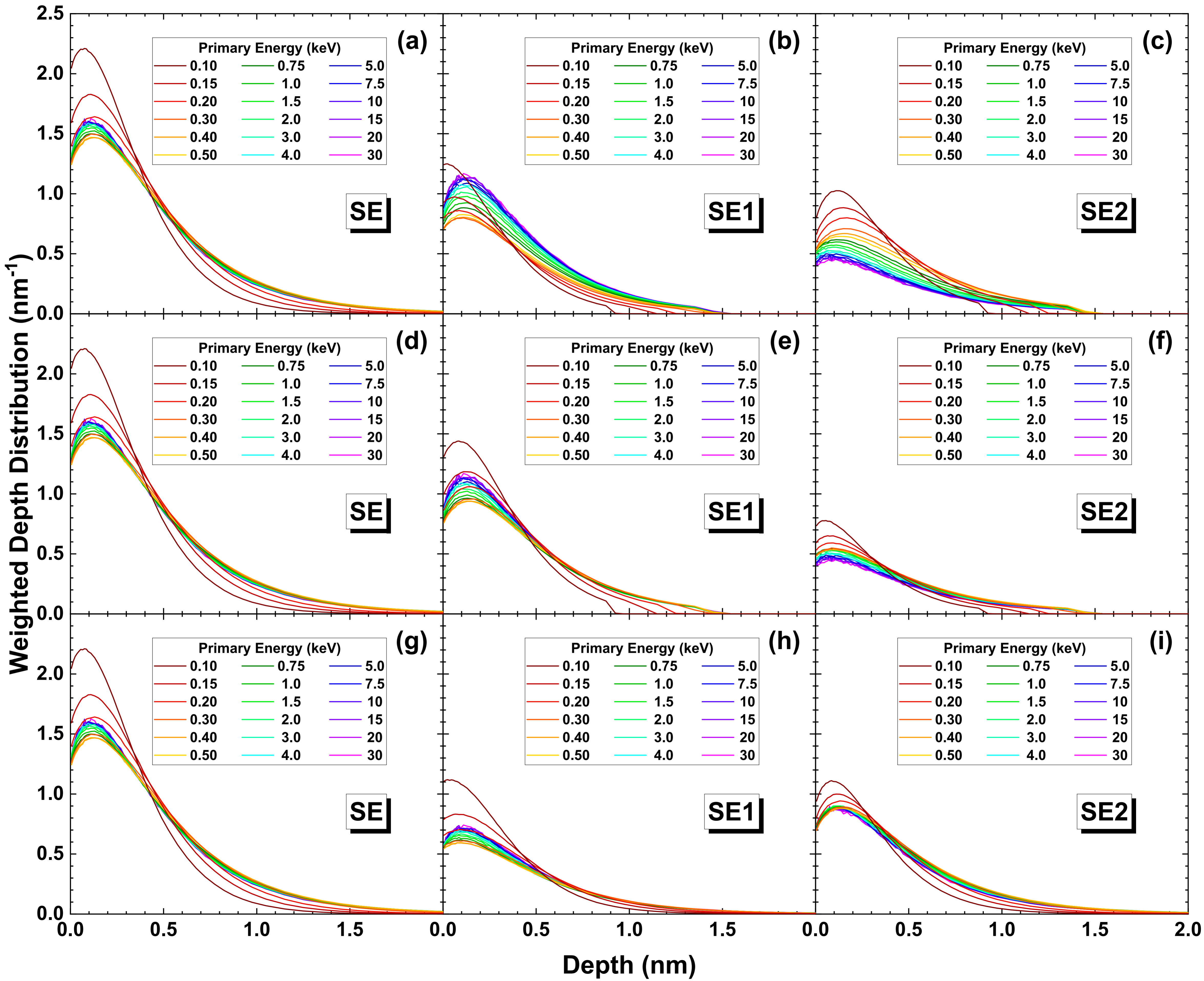

Supplement: Supplementary file 2 — Supporting File 2: advs73849‐sup‐0002‐FigureS1‐S11.zip. [file ADVS-13-e16341-s001.zip › Supplementary Figure 7.pdf]

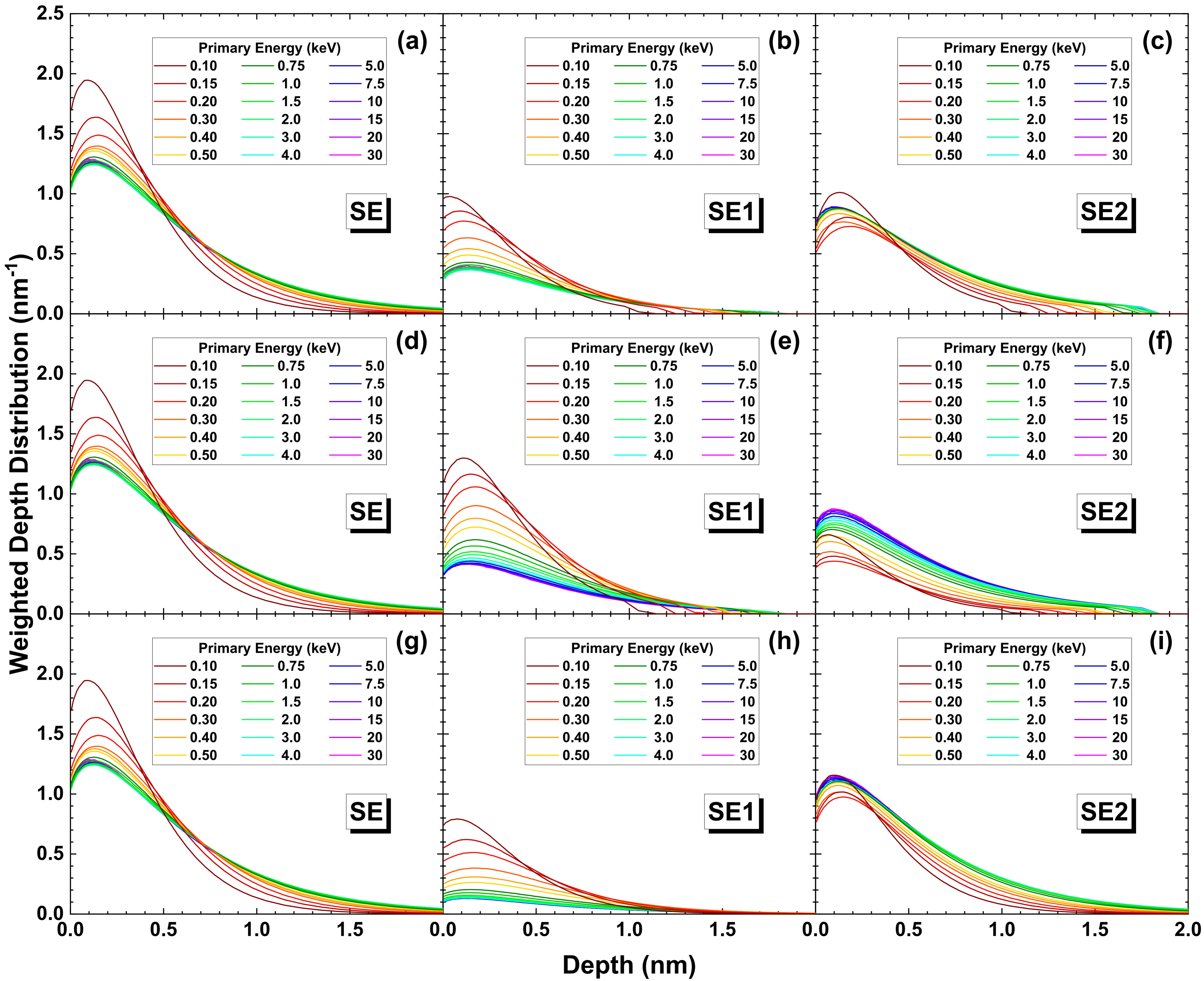

Supplement: Supplementary file 2 — Supporting File 2: advs73849‐sup‐0002‐FigureS1‐S11.zip. [file ADVS-13-e16341-s001.zip › Supplementary Figure 8.pdf]

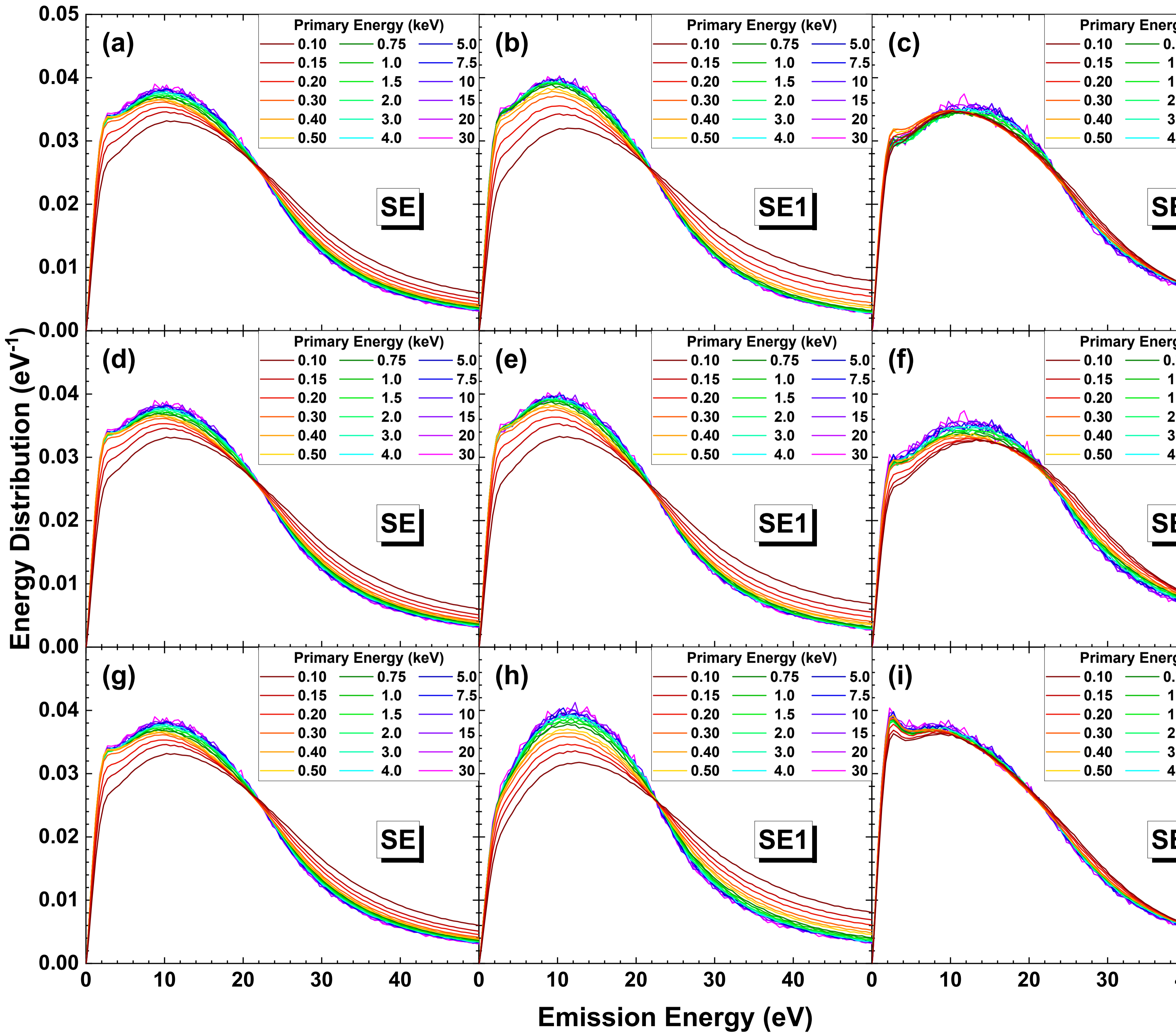

Supplement: Supplementary file 2 — Supporting File 2: advs73849‐sup‐0002‐FigureS1‐S11.zip. [file ADVS-13-e16341-s001.zip › Supplementary Figure 9.pdf]
